# TED1 Promotes the Assembly of SCF^D3^‐D14‐D53 to Reinforce Strigolactone Signaling in Rice

**DOI:** 10.1002/advs.77062

**Published:** 2026-09-09

**Authors:** Sheng Luo, Chuanyin Wu, Miao Feng, Wenfan Luo, Rong Miao, Jinsheng Qian, Fan Wang, Chunlei Zhou, Bojuan Liu, Jiale Shao, Ziqi Xun, Yehui Xiong, Zhichao Zhao, Cailin Lei, Shanshan Zhu, Yulong Ren, Xin Wang, Xiuping Guo, Haiyang Wang, Zichao Li, Zhijun Cheng, Qibing Lin, Jianmin Wan

**Affiliations:** ^1^ State Key Laboratory of Crop Gene Resources and Breeding Institute of Crop Sciences Chinese Academy of Agricultural Sciences Beijing China; ^2^ State Key Laboratory of Agrobiotechnology/Beijing Key Laboratory of Crop Genetic Improvement College of Agronomy and Biotechnology China Agricultural University Beijing China; ^3^ State Key Laboratory For Crop Genetics and Germplasm Enhancement Jiangsu Plant Gene Engineering Research Center Nanjing Agricultural University Nanjing China

**Keywords:** gene evolution, rice, strigolactone signaling pathway, *tiller enhancer and dwarf 1*, ubiquitin‐mediated degradation

## Abstract

Strigolactones (SLs) are a class of terpenoid lactone hormones that repress plant branching, such as tillering in rice. In rice (*Oryza sativa* L.), SLs promote the assembly of the SCF^D3^‐D14‐D53 complex, which triggers the ubiquitination and degradation of the SL signaling repressor D53, thereby activating downstream responses. Here, we identify TED1, a scaffold subunit of the SWR1‐like chromatin remodeling complex, as a critical positive regulator of SL signaling that facilitates the assembly of the SCF^D3^‐D14‐D53 complex. The *ted1* mutants exhibit reduced sensitivity to the SL analogs 5‐deoxystrigol (5DS) and *rac*‐GR24, resulting in increased tillers and dwarfism. We demonstrate that TED1 interacts with D53 independently of SLs and with D14 to enhance the interaction between D14 and D53 or D3 in a SL‐dependent manner, thereby efficiently promoting the ubiquitination and degradation of D53. Furthermore, we show that beyond its canonical role in chromatin remodeling function, the C‐terminal extension of TED1 homologs has evolved a new key function in reinforcing SL signaling, which may represent a key innovation underlying the enhancement of apical dominance in higher plants.

## Graphical abstract

This study identifies TED1 as a positive regulator of strigolactones (SL) signaling in rice. The *ted1* mutants display dwarfism and increased tillering with significantly reduced SLs sensitivity. Through its longer C‐terminal region, TED1 interacts with D53 and D14 to promote the assembly of SCF^D3^‐D14‐D53, thereby facilitating the efficient ubiquitination and degradation of D53 to suppress tillering.

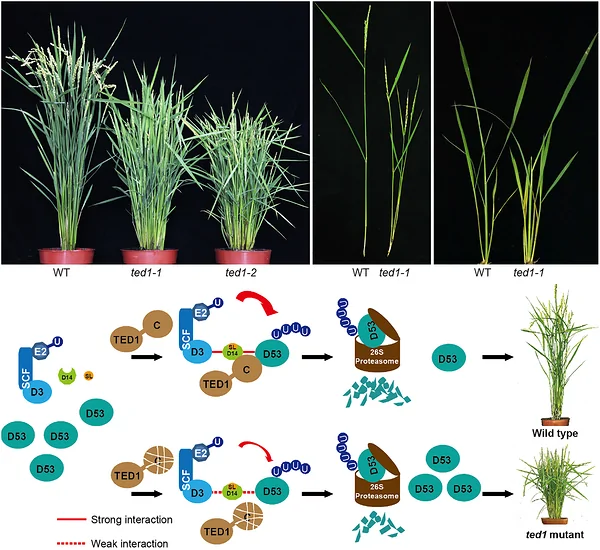

## 1 Introduction

Strigolactones (SLs) are carotenoid‐derived terpenoid lactones originally identified for their roles in stimulating seed germination of the parasitic plant witchweed (*Striga lutea* L.) [1] and inducing hyphal branching in arbuscular mycorrhizal fungi [2]. They were subsequently recognized as hormones that can inhibit branching in higher plants [3, 4]. The biosynthetic pathway and core signaling components of SLs have been well characterized. Genes involved in the SL pathway are designated as *DWARF* (*D*) in rice, *MORE AXILLARY GROWTH* (*MAX*) in *Arabidopsis*, *RAMOSUS* (*RMS*) in pea, and *DECREASED APICAL DOMINANCE* (*DAD*) in petunia. In the biosynthesis of SLs, *D27*/*AtD27* encode a β‐carotene isomerase that transforms all‐*trans*‐β‐carotene into 9‐*cis*‐β‐carotene [5, 6, 7]; *D17*/*MAX3*/*RMS5*/*DAD3* encode a carotenoid cleavage dioxygenase that catalyzes 9‐*cis*‐β‐carotene to form 9‐*cis*‐β‐10’‐carotene [6, 8, 9, 10, 11, 12, 13]; *D10*/*MAX4*/*RMS1*/*DAD1* encode a carotenoid cleavage dioxygenase that catalyzes 9‐*cis*‐β‐10’‐carotene to form carlactone (CL) [6, 14, 15, 16]; *MAX1*/*PhMAX1* encode a member of the cytochrome P450 CYP711 subfamily that catalyzes carlactone to carlactonoic acid (CLA) [17, 18, 19]. Furthermore, some other genes are involved in catalyzing CL and CLA into different types of SLs. CLAMT catalyzes the conversion of CLA to methyl carlactonoate (MeCLA) [20]. Then, LBO, a 2‐oxoglutarate‐dependent dioxygenase, converts MeCLA into hydroxymethyl carlactonoate (1'‐OH‐MeCLA) [21, 22]. For SL signaling, the α/β‐fold hydrolase D14 (and its orthologs RMS and DAD2) functions as the SL receptor [23, 24, 25, 26, 27, 28, 29, 30, 31]. *D3/MAX2/RMS4* encode an F‐box protein that, with Skp1 and Cullin, forms an SCF (Skp1‐Cullin‐F‐box) type E3 ubiquitin ligase [4, 32, 33, 34, 35, 36, 37, 38, 39]. D53/SMXL6, 7, 8 proteins act as repressors of SL signaling. Upon SL perception, D14/AtD14/RMS3/DAD2 hydrolyze SLs and facilitate the assembly of the SCF^D3^‐D14‐D53 complex, promoting the ubiquitination and proteasomal degradation of D53 [40, 41, 42, 43, 44], thus initiating downstream responses [45, 46, 47, 48].

ATP‐dependent chromatin remodeling complexes alter chromatin structure by modulating DNA‐histone interactions. These complexes fall into four subfamilies: Imitation Switch (ISWI), Chromodomain Helicase DNA‐binding (CHD), Switch Defective/Sucrose non‐fermentable (SWI/SNF), and Inositol Requiring 80 (INO80) [49, 50]. They regulate various chromatin remodeling activities, including nucleosome spacing, sliding or eviction, histone variant exchange, genome stability, and DNA repair [49, 50]. Within the INO80 subfamily, two major complexes exist: INO80 and SWR1. The SWR1 complex specifically replaces histone H2A‐H2B histone dimers with H2A.Z‐H2B [48, 50]. Acetylation of H2A.Z has been associated with transcriptional activation, whereas its mono‐ubiquitination generally correlates with transcriptional repression [51, 52, 53].

*PHOTOPERIOD‐INDEPENDENT EARLY FLOWERING1* (*PIE1*), the *Arabidopsis* homolog of the yeast (*Saccharomyces cerevisiae*) *SWR1* gene, encodes the scaffold subunit SWR1 of the SWR1 complex. Mutations in *PIE1* lead to reduced expression of *FLOWERING LOCUS C* (*FLC*), a key floral repressor, thereby resulting in an early flowering phenotype [54, 55, 56]. The *pie1* mutant also exhibits pleiotropic phenotypes, including serrated leaves, weakened apical dominance with excessive branching, reduced fertility, and altered plant stature [54, 55, 56], suggesting that *PIE1* is involved in multiple developmental processes. However, the precise mechanisms by which *SWR1*‐like genes regulate plant branching remain unclear.

In this study, we identified a rice mutant, *tiller enhancing and dwarf 1‐1* (*ted1‐1*), from a CRISPR/Cas9‐generated mutant library. We found that *TED1*, encoding a SWR1‐like protein, is the causal gene underlying the *ted1‐1* phenotype. Our biochemical analysis revealed that TED1 interacts with D53 independently of *rac*‐GR24, and with D14 in the presence of *rac*‐GR24 to strengthen the interaction between D14 and either D53 or D3, thus ensuring the efficient ubiquitination and degradation of D53 via the Ubiquitin‐26S proteasome pathway. Further analysis revealed that, besides its established role in chromatin remodeling, TED1‐like proteins have acquired a crucial new regulatory function—reinforcing SL signaling via the C‐terminal extension—likely for enhancing apical dominance during plant type evolution.

## 2 Results

### 2.1 Phenotypic Characterization of the *ted1‐1* Mutant

**FIGURE 1:**
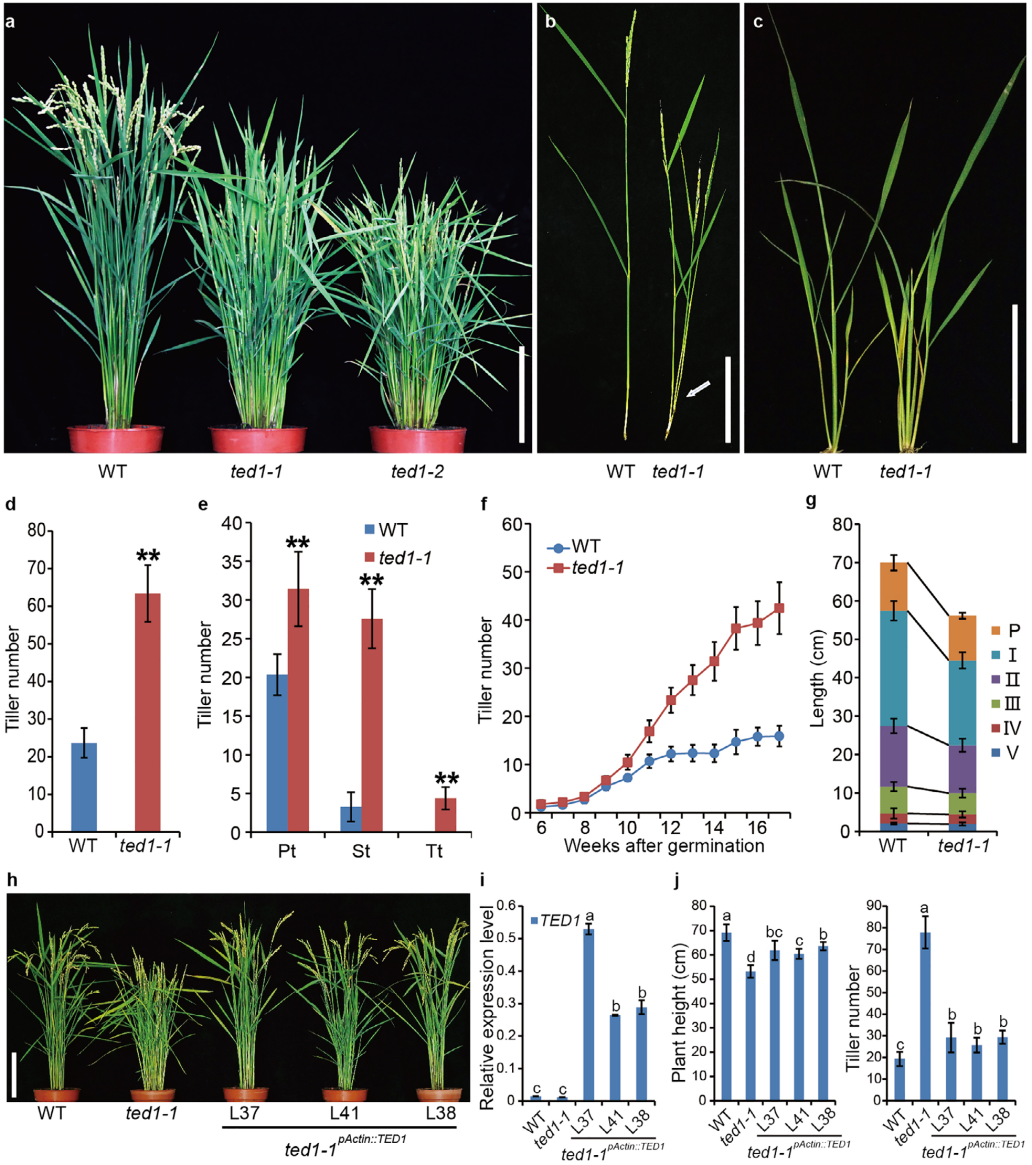
The *ted1* mutants display a dwarf phenotype with increased tillering. (a) Phenotypic comparison between wild‐type (WT), *ted1‐1* and *ted1‐2* plants at the seed‐filling stage. Scale bar: 10 cm. (b) *ted1‐1* exhibits additional tillers emerging from un‐elongated internodes (white arrow) compared to WT. Scale bar: 15 cm. (c) Phenotypic comparison between wild‐type (WT) and *ted1‐1* plants at the 5‐week‐old seedling stage. Scale bar: 15 cm. (d) Comparison of total tiller numbers between WT and *ted1‐1*. Values are means ± s.d. (*n* > 20). *P*‐values are from two‐sided Student's *t*‐tests (^*^, *p* < 0.05; ^**^, *p* < 0.01). (e) Comparison of primary tillers (Pt), secondary tillers (St), and tertiary tillers (Tt) between WT and *ted1‐1*. Values are means ± s.d. (*n* > 10). *P*‐values are from two‐sided Student's *t*‐tests (^*^, *p* < 0.05; ^**^, *p* < 0.01). (f) Tiller numbers increase along plant development in WT and *ted1‐1*. Values are means ± s.d. (*n* > 20). (g) Comparison of panicle length (P) and internode length (I, II, III, IV, V) between WT and *ted1‐1*. Values are means ± s.d. (*n* > 20). (h) Overexpressing *TED1* rescued the *ted1‐1* mutant phenotype. Phenotype of WT, *ted1‐1*, and three independent *pActin::TED1* transgenic lines in the *ted1‐1* mutant background. Scale bar: 15 cm. (i) Relative expression level of *TED1* in WT, *ted1‐1*, and three *pActin::TED1* transgenic lines in the *ted1‐1* background. Values are means ± s.d. (*n* = 3). *P*‐values are from one‐way ANOVA tests with Tukey's correction (significant difference between different letters). (j) Plant height and tiller numbers of WT, *ted1‐1*, and three *pActin::TED1* transgenic lines in the *ted1‐1* background. Values are means ± s.d. (*n* > 12). *P*‐values are from one‐way ANOVA tests with Tukey's correction (significant difference between different letters).

Plant architecture determines plant type and directly affects grain production in rice. To identify novel genes involved in the regulation of plant architecture, we generated a CRISPR/Cas9 mutant library in the *Japonica* rice cultivar Kitaake. From this library, we identified a mutant exhibiting dwarfism and increased tillering, which we designated *ted1* (*tiller enhancing and dwarf 1*, hereafter referred to as *ted1‐1*). To confirm this, we identified another four mutants with different mutation patterns, *ted1‐2* to *ted1‐5* (Figure 1a, Figure S1). All five mutants exhibited dwarfism and increased tillering (Figure S1a,c). Among them, *ted1‐1* and *ted1‐2* were derived from the same sgRNA targeting site, while *ted1‐3 to ted1‐5* were derived from a different sgRNA target site (Figure S1b). Genetic analysis showed that F_1_ progeny from reciprocal crosses between wild‐type (WT) and *ted1‐1* displayed a WT‐like phenotype (Figure S2a,b), while the F_2_ generation segregated in an approximately 3:1 ratio of WT to mutant individuals (Figure S2c), indicating that *ted1‐1* is controlled by a single recessive nuclear gene.

The *ted1‐1* mutant was selected as a representative mutant for further analyses. We found that *ted1‐1* exhibited significantly increased tillers, which mainly resulted from more secondary tillers on the primary tillers at the seedling stage (Figure 1b,c). At the mature stage, the total number of tillers in *ted1‐1* was approximately three times that in WT, owing to the increases in primary (Pt), secondary (St), and tertiary (Tt) tillers (Figure 1d,e). Developmental analysis revealed that the *ted1‐1* mutant consistently produced more tillers than WT from the seedling stage to the maturity stage (Figure 1f). The dwarfism observed in *ted1‐1* was attributed to reduced lengths of the panicle and internode I to III (Figure 1g, Figure S3).

To further validate that the dwarf and high‐tillering phenotype was caused by mutation in *TED1*, we overexpressed the full‐length *TED1* coding sequence under the control of the rice *Actin* promoter in the *ted1‐1* background. Transgenic lines carrying the *pActin::TED1* construct restored the mutant phenotype to that of WT (Figure 1h–j). These results verified *TED1* as the gene responsible for the *ted1‐1* mutant phenotype.

### 2.2 *TED1* Encodes a SWR1‐Like Protein

Sequence analysis revealed that *TED1* shares homology with *PIE1* in *Arabidopsis thaliana* and *SWR1* in yeast, both of which encode ATP‐dependent chromatin remodeling proteins belonging to the Ino80 subfamily. Protein sequence alignment showed that TED1 contains three conserved domains (HSA, DEXDc, and HELICc) as well as two Coiled‐coil domains (Figure S4).

The full‐length coding sequence of *TED1* spans 6135 nucleobases and encodes a protein of 2044 amino acids. Due to its large size, all attempts to express full‐length TED1 using various systems, including rice protoplasts, tobacco (*Nicotiana benthamiana*) leaves, *E*. coli, yeast, and the Baculovirus expression system, were unsuccessful. To overcome this limitation, we employed two strategies. First, we divided TED1 into several segments based on its domain structure, and found that the resulting truncated proteins were well expressed. Second, we codon‐optimized the full‐length coding sequence of *TED1*, which significantly improved its expression efficiency (Table S1).

**FIGURE 2:**
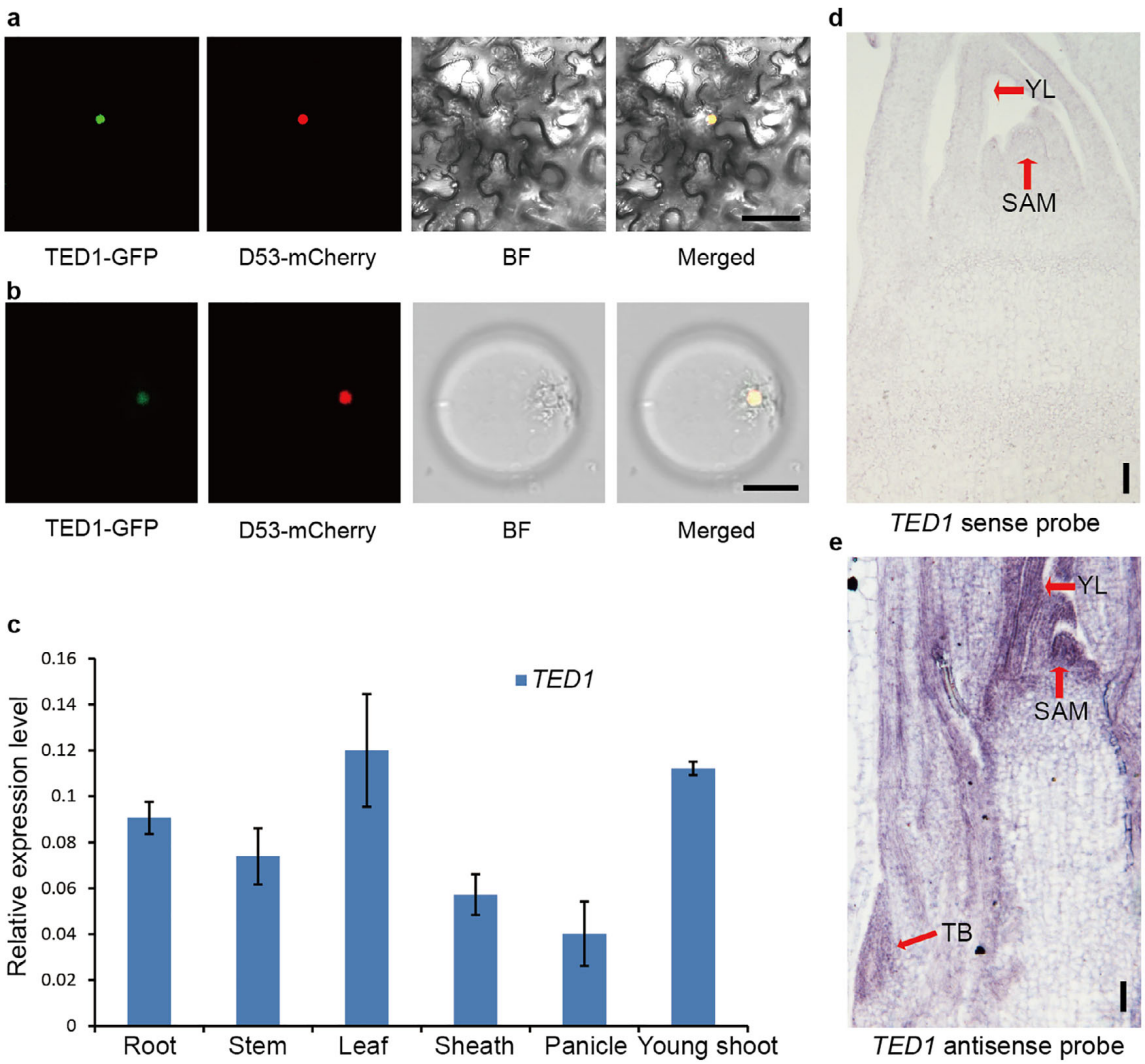
Subcellular localization and expression pattern of the *TED1* gene. (a, b) TED1‐GFP was localized in the nucleus of tobacco (*N. benthamiana*) leaves (a) and rice protoplasts (b). D53‐mCherry was used as a nucleus marker. BF, bright field. These experiments used the optimized CDS sequence of *TED1*. Scale bars: 50 µm (a), 10 µm (b). (c) Relative expression level of *TED1* in Root, Stem, Leaf, Sheath, Panicle, and Young Shoot. The rice ubiquitin gene served as the internal control. Error bars represent variation among three biological replicates. Values are means ± s.d. (*n* = 3). (d, e) RNA in situ hybridization revealed the expression of *TED1* in the shoot apical meristem (SAM), tiller bud (TB), and young leaf (YL) (e). The *TED1* sense probe was used as the negative control (d). Scale bars: 100 µm (d, e).

Transient expression of TED1 in tobacco leaves and rice protoplasts showed robust nuclear localization (Figure 2a,b), consistent with the nuclear functions of SWR1 and PIE1. Real‐time Quantitative PCR (RT‐qPCR) analysis revealed broad expression of *TED1* in roots, stems, leaves, sheaths, panicles, and young shoots (Figure 2c). RNA in situ hybridization further confirmed *TED1* expression in the shoot apical meristem (SAM), tiller buds (TB), and young leaves (YL) (Figure 2d,e), consistent with the dwarfism and enhanced tillering phenotypes observed in *ted1* mutants.

### 2.3 TED1's Subtle Role in Tiller Number Regulation via H2A.Z‐Mediated Chromatin Remodeling

Given the established role of the SWR1 complex in exchanging canonical H2A‐H2B histone dimers with H2A.Z‐H2B, we investigated potential interaction between TED1 and H2A.Z variants in rice. BLASTp searches identified three H2A.Z homologs: H2AV1, H2AV2, and H2AV3. To test their interactions, TED1 was divided into three segments: TED1‐N (amino acids 1 to 500; containing the HSA and one Coiled‐coil domain), TED1‐M (amino acids 240 to 1,010; containing the DEXDc domain), and TED1‐C (amino acids 986 to 2,044; containing the HELICc and the second Coiled‐coil domain). Yeast two‐hybrid (Y2H) assays revealed that TED1‐N interacted with all three H2A.Z variants (Figure S5), suggesting a potential role for TED1 in H2A.Z‐associated chromatin remodeling.

To assess the functional relevance of these interactions, we generated single, double, and triple mutants of *H2AV1*, *H2AV2*, and *H2AV3* using CRISPR/Cas9. Similar to *ted1‐1*, all mutants exhibited significantly reduced plant height compared to WT. However, only *h2av1*, *h2av1/h2av2*, and *h2av2/h2av3* showed a modest increase in tiller number, whereas *h2av2*, *h2av3*, *h2av1/h2av3*, and *h2av1/h2av2/h2av3* displayed tiller numbers comparable to WT (Figure S6).

Overall, these findings suggest that TED1 exerts only a subtle influence on tiller number regulation through H2A.Z‐mediated chromatin remodeling.

### 2.4 The Sensitivity of *ted1* to SL is Significantly Decreased

Given the phenotypic similarity between *ted1* mutants and those defective in the SL pathway, we investigated whether *TED1* functions in this pathway. RT‐qPCR analysis revealed reduced expression levels of *D53*, *D3*, *D14*, *FC1*, *D27*, and *D17* in *ted1‐1*, whereas *D10* expression was significantly upregulated compared to WT (Figure S7a). This pattern mirrors that observed in *d53*, *d14*, and *d27* mutants, where *D10* is also upregulated [41]. Conversely, *TED1* expression was decreased in multiple SL pathway mutants, including *d3*, *d14*, *d27*, *d17*, *d10*, and *d53* (Figure S7b). In addition, similar to the induced expression of some genes in the SL pathway by phosphate (Pi) deficiency [57], the expression of *TED1* is also induced by Pi deficiency (Figure S8). Together, these findings suggest that *TED1* may participate in the SL pathway.

**FIGURE 3:**
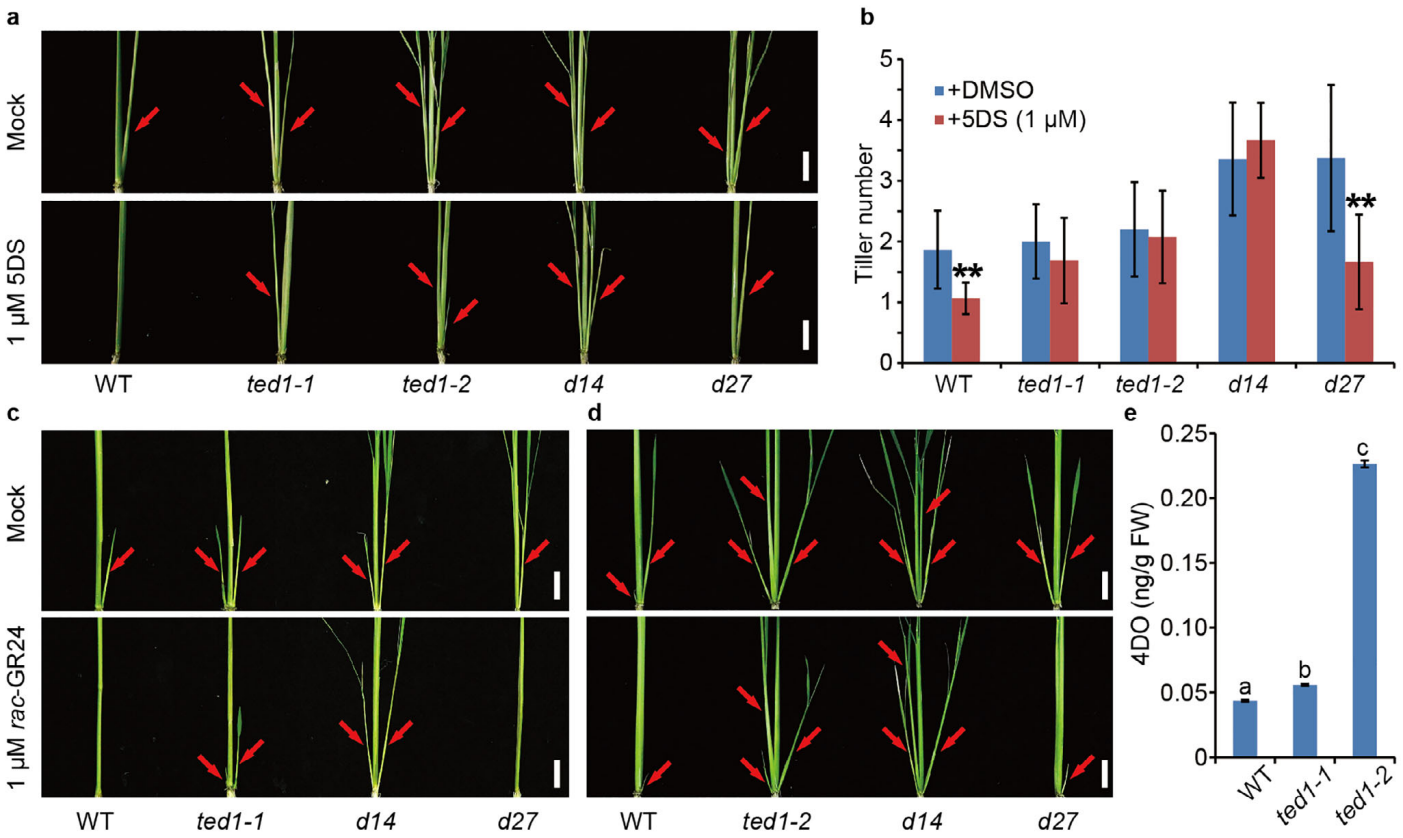
The sensitivity of *ted1* mutants to SLs is significantly reduced. (a, b) The sensitivity of *ted1‐1* and *ted1‐2* to 5DS (a) and the number of tillers in different materials (b). Red arrows mark tillers. The asterisks indicate the significance of the difference between the mutants and the wild type. The concentration of 5DS in the experimental group was 1 µM. Scale bars: 2 cm (a). Values are means ± s.d. (b, *n* > 20). *P*‐values are from two‐sided Student's *t*‐tests (b, ^*^, *p* < 0.05; ^**^, *p* < 0.01). (c, d) The *ted1‐1* mutant (c) exhibits hyposensitivity, while *ted1‐2* (d) shows almost complete insensitivity to *rac*‐GR24 treatment. Red arrows mark tillers. The concentration of *rac*‐GR24 in the experimental group was 1 µM. Scale bars: 2 cm (c, d). (e) Comparison of root exudates 4DO between WT, *ted1‐1*, and *ted1‐2* by Liquid chromatography‐tandem mass spectrometry (LC‐MS/MS). ng/g FW, ng per gram fresh weight. Values are means ± s.d. (*n* = 3). *P*‐values are from one‐way ANOVA tests with Tukey's correction (significant difference between different letters).

To assess the functional relevance of TED1 in the SL pathway, we examined the responses of WT, *ted1‐1, ted1‐2*, *d14* (the mutant of the SL receptor gene), and *d27* (a mutant of the SL biosynthesis gene) to the SL analog (±)‐5‐deoxystrigol (5DS). Application of 1 µM 5DS failed to inhibit tiller outgrowth in *d14*, but significantly suppressed tillering in WT and *d27*. Similar to *d14*, *ted1‐1* and *ted1‐2* showed only mild suppression of tiller outgrowth (Figure 3a,b). Treatment with another SL analog, *rac*‐GR24, partially suppressed tillering in *ted1‐1*, but had little to no effect on *ted1‐2* (Figure 3c,d). Furthermore, root exudates from *ted1‐1* and *ted1‐2* contained elevated levels of 4‐deoxyorobanchol (4DO), a native SL, compared to WT (Figure 3e, Figure S9a). Moreover, the 4DO content in the root exudates of *ted1‐2* is higher than that of *ted1‐1*, which may be related to the more severe phenotype of *ted1‐2* (Figure 3e). We also attempted to detect the orobanchol (ORO) content in the root exudates, but we did not detect any ORO, possibly due to its low abundance in the Kitaake background (Figure S9b). These results indicate that *ted1‐1* is SLs‐hyposensitive, whereas *ted1‐2* is SLs‐insensitive, supporting the notion that *TED1* plays a critical role in SL signaling.

Given the reduced expression of *D14* and *D3* in *ted1‐1* (Figure S7a), we hypothesized that *TED1* might regulate SL signaling by modulating their expression levels. However, the overexpression of *D14* in the *ted1‐1* background did not rescue the mutant phenotype (Figure S10). Moreover, all three independent Western blots showed that there were no significant differences in D3 and D14 protein levels between WT and *ted1‐1* (Figure S11a–c). We further examined the protein level of D14 in transgenic lines overexpressing *D14* in the *ted1‐1* background, and found that the overexpression of *D14* can substantially increase D14 protein levels (Figure S11d). These results suggest that *TED1* does not control the SL pathway by altering the abundance of D14 and D3 proteins.

### 2.5 TED1 Interacts With D14 and D53

**FIGURE 4:**
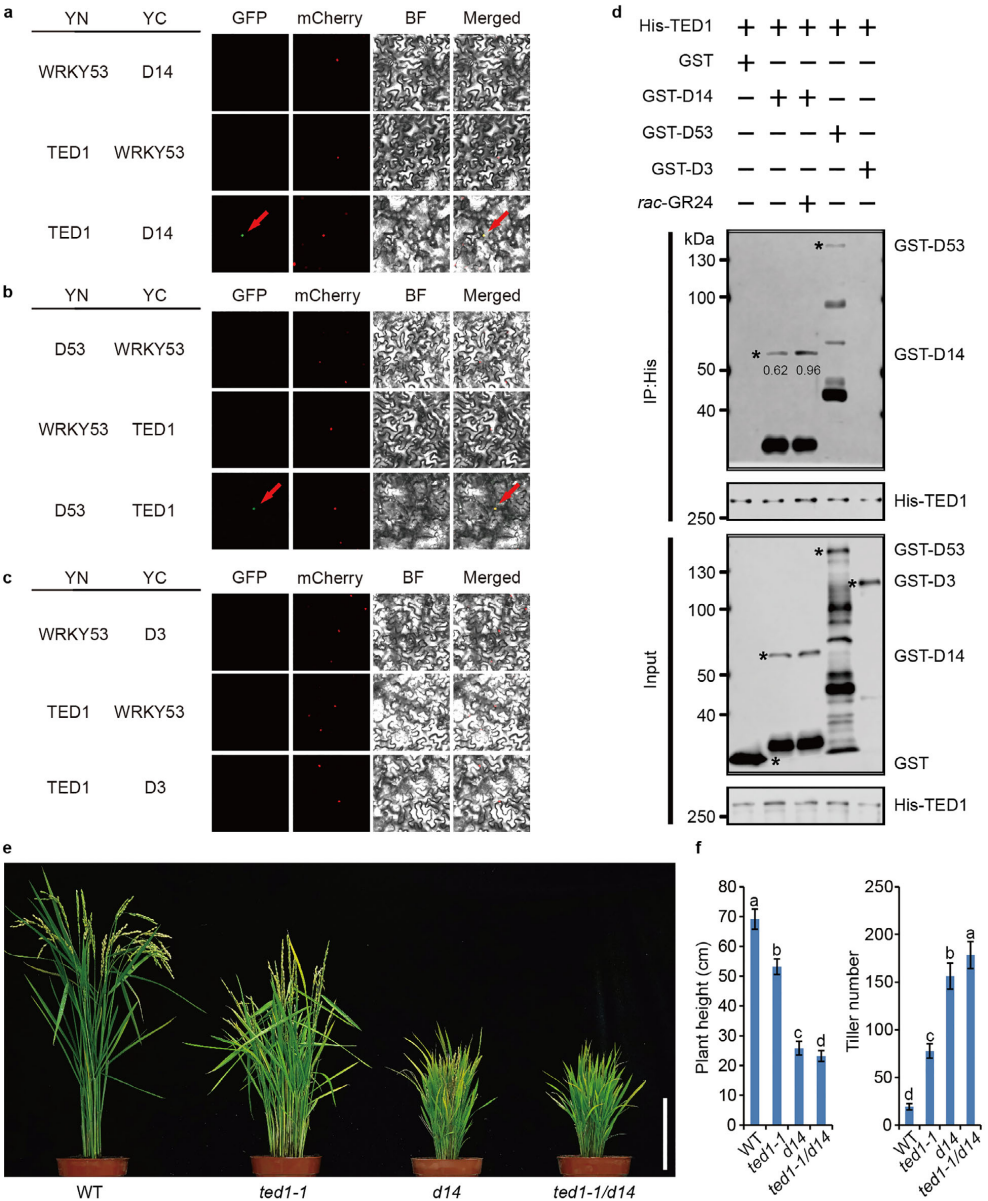
TED1 regulates SL signaling by interacting with D14 and D53. (a, b, c) BiFC assays showing protein‐protein interactions in *N. benthamiana* leaf epidermal cells. TED1 interacts with D14 (a). TED1 interacts with D53 (b). No interaction was detected between TED1 and D3 (c). These experiments used the optimized CDS sequence of *TED1*. YN and YC indicate the N‐terminal and C‐terminal fragments of GFP, respectively. GFP and mCherry fluorescence, bright‐field (BF) images, and merged images are shown. WRKY53 was used as the negative control. mCherry showed the position of the nucleus, with red arrows pointing to the fluorescent signal of the interaction. (d) Pull‐down analysis of the in vitro interactions between TED1 and D14, D53, and D3. His‐TED1, GST, GST‐D14, GST‐D53, and GST‐D3 were individually expressed in *Escherichia coli* and purified. Pull‐down assays were performed using beads conjugated with His‐TED1. The results showed that GST‐D14 and GST‐D53 were pulled down by His‐TED1, whereas GST‐D3 was not pulled down by His‐TED1 alone. Furthermore, *rac*‐GR24 enhanced the interaction between His‐TED1 and GST‐D14. The asterisk indicates the target protein bands. Numbers represent the protein band intensities of GST‐D14 relative to the His‐TED1 protein band after IP. (e, f) Plant phenotype of WT, *ted1‐1*, *d14*, and *ted1‐1*/*d14* double mutant. Scale bar: 15 cm (e). Values are means ± s.d. (f, *n* > 14). *P*‐values are from one‐way ANOVA tests with Tukey's correction (f, significant difference between different letters).

To explore alternative mechanisms, we first tested potential interactions between TED1 and key SL signaling components (D14, D3, and D53) using a bimolecular fluorescence complementation (BiFC) assay. Both D14 and D53 were found to interact with TED1 in the nucleus (Figure 4a–c), and all three independent pull‐down assays confirmed the TED1‐D14 interaction, which was enhanced by *rac*‐GR24 treatment (Figure 4d, Figure S12). To test their genetic interaction, a *ted1‐1 d14* double mutant was generated. The double mutant exhibited a phenotype more similar to *d14* than *ted1‐1*, with shorter stature and more tillers (Figure 4e,f), suggesting that *TED1* acts predominantly in the same pathway as *D14* to regulate plant height and tillering.

To further map the interaction domains, TED1 was divided into segments based on domain structure. The Y2H assays revealed that only the C‐terminal portion (TED1‐C) interacted with D14 in a *rac*‐GR24‐dependent manner, and neither TED1‐N nor TED1‐M showed any interaction with D14 (Figure S13a). This interaction was further validated through Co‐immunoprecipitation (Co‐IP) assays (Figure S13b). To fine‐map the interaction region, TED1‐C was subdivided into six fragments (Figure S14a), and Y2H assays revealed that C2, C5, and C6 fragments could interact with D14 in the presence of *rac*‐GR24. C2 corresponds to the HELICc domain, and C4 includes the Coiled‐coil domain (Figure S14b).

We next investigated TED1's interaction with D53. TED1 was split into TED1‐NM (amino acids 1 to 1,010) and TED1‐C (amino acids 986 to 2,044) (Figure S15a). Co‐IP assays in tobacco leaves revealed that TED1‐C, but not TED1‐NM, interacted with D53 (Figure S15b,c). No interactions were detected between either TED1 fragment and D3 (Figure S15d,e). These results demonstrate that TED1 regulates SL signaling by interacting with both D14 and D53 through its C‐terminal domain, and that the TED1‐D14 interaction is SL‐dependent.

To investigate the cause of the phenotypic differences between *ted1‐1* and *ted1‐2*, we compared the two different C‐terminal truncated protein sequences (TED1‐C‐MT1 derived from *ted1‐1* and TED1‐C‐MT2 from *ted1‐2*) generated by the premature stop codons in the two mutants. We found that TED1‐C‐MT1 and TED1‐C‐MT2 are nearly identical, except that TED1‐C‐MT2 contains two additional isoleucine (Ile, I) residues at the C‐terminus compared to TED1‐C‐MT1 (Figures S14a and S16a). Yeast two‐hybrid results showed that both TED1‐C‐MT1 and TED1‐C‐MT2 can interact with D14 in a *rac*‐GR24‐dependent manner, and the interaction between TED1‐C‐MT2 and D14 is weaker than that between TED1‐C‐MT1 and D14. However, neither mutant form could interact with D53 or D3 (Figure S16b). These results suggest that the function of TED1 in regulating SL signaling is more severely compromised in *ted1‐2* than in *ted1‐1*, which is consistent with the more severe phenotype observed in *ted1‐2* than in *ted1‐1*.

### 2.6 TED1 Promotes the Ubiquitination and Degradation of D53 Protein

**FIGURE 5:**
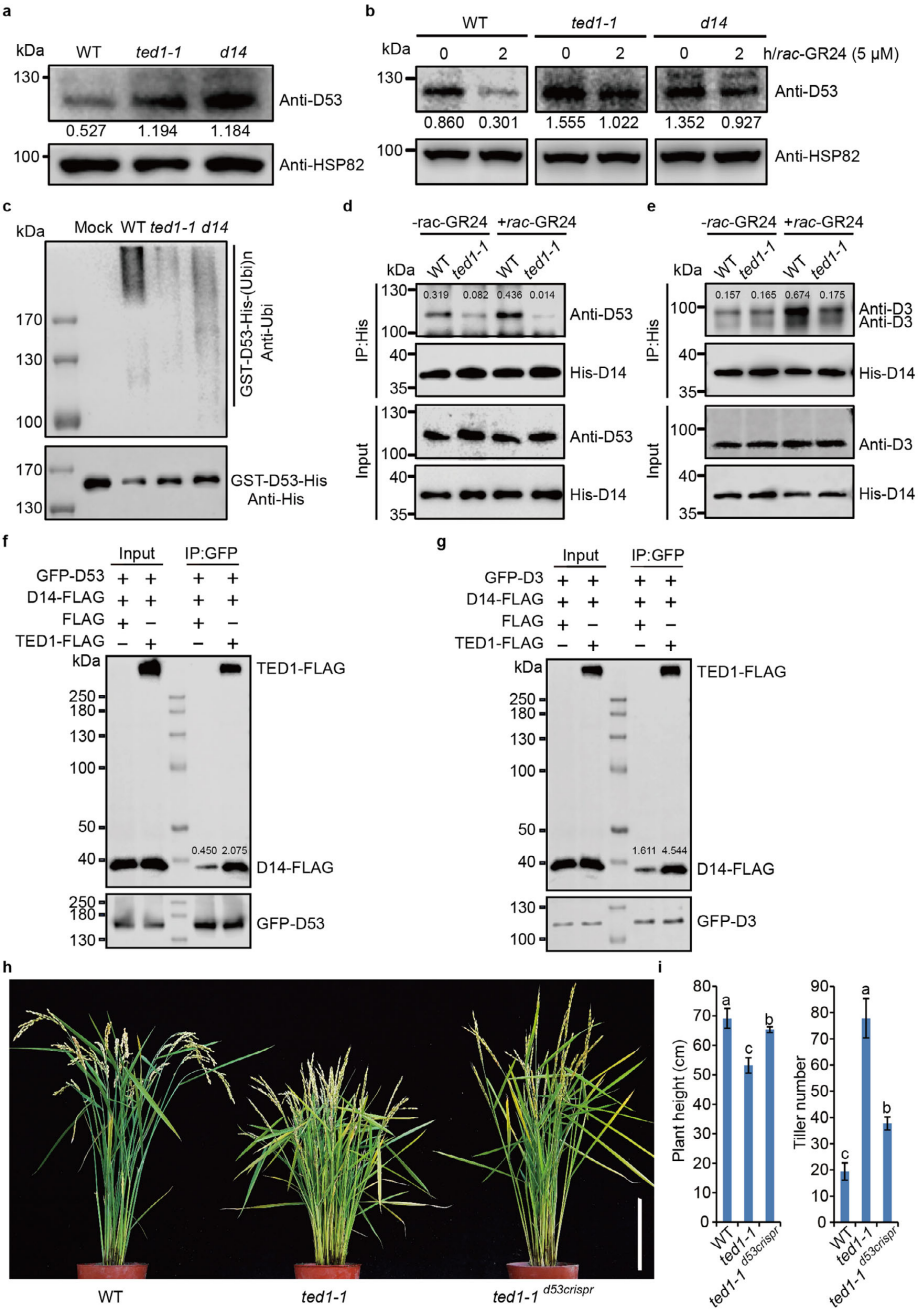
TED1 is essential for the ubiquitination and degradation of the D53 protein. (a) *ted1‐1* and *d14* mutants exhibit higher accumulation of D53 protein compared to the WT, with HSP82 serving as the internal control. Numbers represent the protein band intensity of D53 relative to the internal control protein HSP82. (b) *rac*‐GR24 promotes D53 degradation in the WT, but not in the *ted1‐1* or *d14* mutants. Numbers represent the protein band intensity of D53 relative to HSP82. (c) In vitro ubiquitination assay demonstrates that GST‐D53‐His is more effectively polyubiquitinated by the WT total proteins compared to *ted1‐1* and *d14* mutant total proteins. (d) Semi‐In vivo pull‐down assay indicates that TED1 is necessary for the efficient enrichment of D53 by His‐D14, especially in the presence of 5 µM *rac*‐GR24. Numbers represent the D53 protein band intensity relative to the His‐D14 protein band after IP. (e) Semi‐In vivo pull‐down assay reveals that TED1 could enhance the enrichment of D3 by His‐D14 in the presence of 5 µM *rac*‐GR24. Numbers represent the D3 protein band intensity relative to the His‐D14 protein band after IP. (f) In vivo Co‐IP analysis demonstrates that the TED1‐FLAG fusion protein promotes the interaction between GFP‐D53 and D14‐FLAG fusion protein in the leaves of *N. benthamiana*. (g) In vivo Co‐IP analysis demonstrates that the TED1‐FLAG fusion protein promotes the interaction between GFP‐D3 and D14‐FLAG fusion protein in the leaves of *N. benthamiana*. (h, i) Plant phenotypes of WT, *ted1‐1*, and *ted1‐1^d35crispr^* double mutant. Scale bar: 15 cm (h). Values are means ± s.d. (i, *n* > 10). *P*‐values are from one‐way ANOVA tests with Tukey's correction (i, significant differences between different letters).

Since the SCF^D3^‐D14 complex targets D53 for ubiquitination and degradation in the presence of SLs [40, 41], we investigated whether TED1 influences this process. Western blot analysis revealed a marked accumulation of the D53 protein in both *ted1‐1* and *d14* compared to WT (Figure 5a, Figure S17a). Furthermore, *rac*‐GR24 treatment significantly promoted D53 degradation in WT but not in the *ted1‐1* and *d14* mutants (Figure 5b, Figure S17b). Our in vitro ubiquitination assays revealed that GST‐D53‐His was efficiently ubiquitinated by total protein extracts from WT, but not from *ted1‐1* or *d14* mutants (Figure 5c). These results indicate that, similar to D14, TED1 plays a key role in promoting the ubiquitination and degradation of D53.

We next tested whether TED1 affects the assembly of the SCF^D3^‐D14‐D53. Semi‐In vivo pull‐down assays demonstrated that His‐D14 pulled down more D53 protein from WT extracts than from *ted1‐1* extracts in the absence of exogenous *rac*‐GR24. The addition of *rac*‐GR24 further enhanced the D14‐D53 interaction in WT, but not in *ted1‐1* extracts (Figure 5d, Figure S17c). Similarly, *rac*‐GR24 promoted the enrichment of D3 by D14 in WT extracts but not in *ted1‐1* extracts (Figure 5e, Figure S17d). Interestingly, in the absence of *rac*‐GR24, His‐D14 pulled down comparable amounts of D3 protein from both WT and *ted1‐1* extracts (Figure 5e, Figure S17d), suggesting that TED1 enhances D14's interactions with both D53 and D3 specifically in the presence of SLs. Additionally, two bands corresponding to D3 were detected in the pull‐down assays, indicating possible post‐translational modifications during the assay (Figure 5e).

Co‐IP assays were performed in *N. benthamiana* leaves co‐expressing GFP‐D53, D14‐FLAG, and TED1‐FLAG (with the FLAG tag alone as a negative control). After an immunoprecipitation assay with GFP beads, the D14‐FLAG signal was substantially enhanced in the presence of TED1‐FLAG compared to the control (Figure 5f, Figure S17e). Similarly, in *N. benthamiana* leaves, co‐expressing GFP‐D3, D14‐FLAG, and TED1‐FLAG (with the FLAG tag alone as a negative control), an immunoprecipitation assay with GFP beads revealed a markedly increased D14‐FLAG signal upon co‐expression of TED1‐FLAG (Figure 5g, Figure S17f). These results indicated that TED1 did promote the interaction between D14 and D3 or between D14 and D53 in planta.

To investigate the genetic relationship between *TED1* and *D53*, we generated *ted1‐1^d53crispr^* double mutants using the CRISPR/Cas9 technology. Strikingly, *ted1‐1^d53crispr^* plants displayed plant height and tiller number comparable to WT, effectively rescuing the *ted1‐1* phenotype (Figure 5h,i). Notably, the partial suppression of *ted1‐1* by *d53* knockout may be due to a closely related gene, *D53‐like*, in rice, and both *D53* (Os11g0104300) and *D53‐like* (Os12g0104300) may need to be knocked out for full phenotypic suppression. Therefore, these findings suggest that *TED1* acts upstream of *D53* in regulating rice plant height and tillering.

### 2.7 The Interaction Between TED1's Homologs and D14's Homologs is Likely Conserved in Plants

**FIGURE 6:**
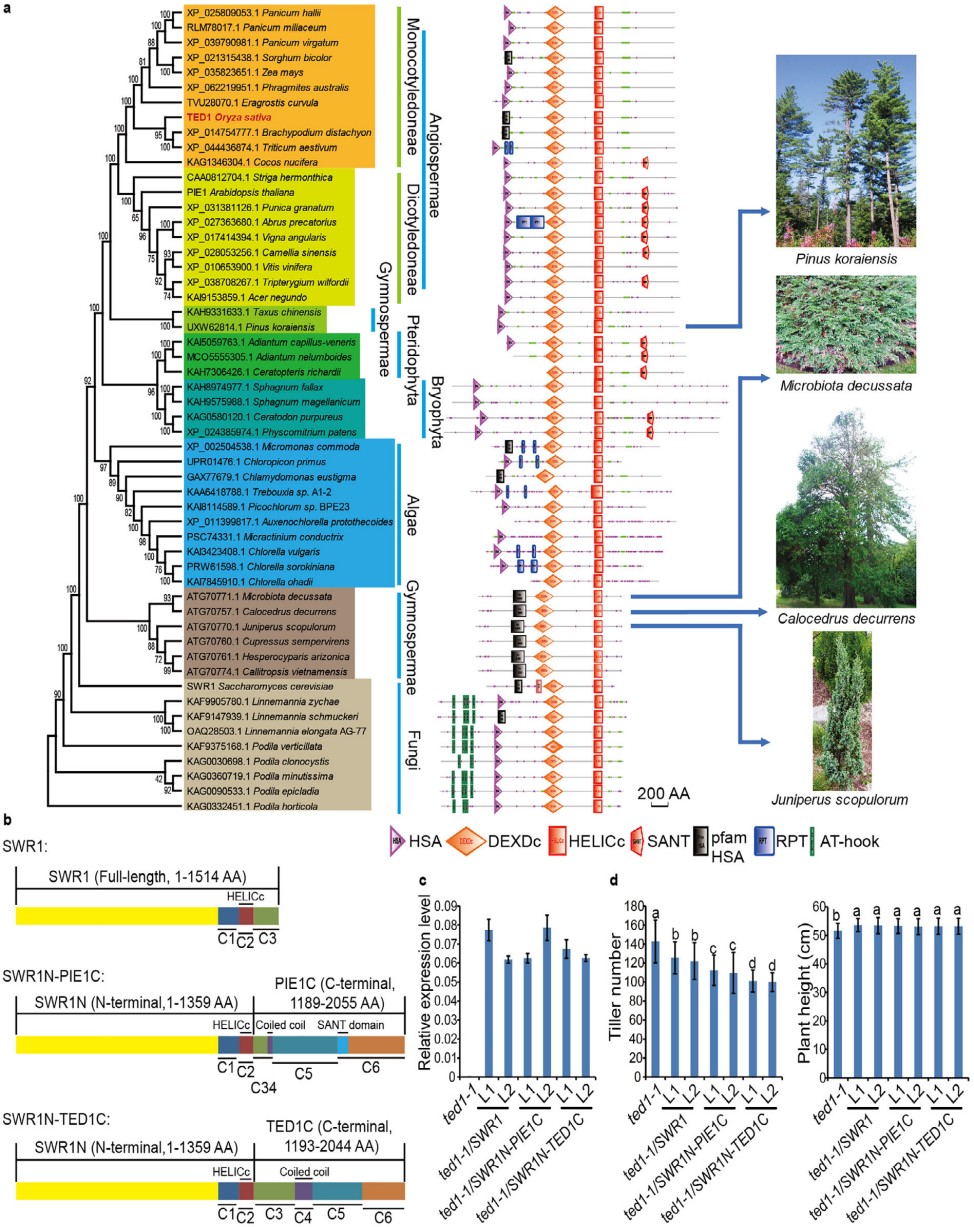
The C‐terminal extension of SWR1 can enhance its repression on rice tillering. (a) The TED1 protein sequence was used for a BLASTp search on NCBI. Protein sequences from various species were selected, and a phylogenetic tree was constructed using MEGA11. The SMART website was utilized to analyze protein structural domains, and the domain names were obtained from the SMART website. The rightest pictures show that *Pinus koraiensis* has a tall tree crown (strong apical dominance), while *Microbiota decussata*, *Calocedrus decurrens*, and *Juniperus scopulorum* display bushy forms with absent or weak apical dominance. Plant images were obtained from the Plant Photo Bank of China (PPBC, https://ppbc.iplant.cn) and are used under license. (b) Schematic diagram of the fusion gene structures of *SWR1N‐PIE1C* and *SWR1N‐TED1C*; the fusion genes were overexpressed in the *ted1‐1* mutant under the control of the Actin promoter. (c) Detection of the expression levels of *SWR1*, *SWR1N‐PIE1C*, and *SWR1N‐TED1C* in the corresponding transgenic plants of the *ted1‐1* mutant, respectively. Values are means ± s.d. (*n* = 3). (d) Statistics of the tiller number and plant height of *ted1‐1* and the transgenic *ted1‐1* plants expressing *SWR1*, *SWR1N‐PIE1C*, and *SWR1N‐TED1C*, respectively. Values are means ± s.d. (*n* > 15). *P*‐values are from one‐way ANOVA tests with Tukey's correction (significant difference between different letters).

To explore whether the TED1‐D14 interaction is evolutionarily conserved, we examined the interaction between *Arabidopsis* homologs of these proteins. PIE1, the *Arabidopsis* homolog of TED1, and AtD14, the homolog of rice D14, were tested for interaction. Like TED1‐C (Figure S14a), the PIE1 C‐terminal region (PIE1‐C) was divided into five fragments. PIE1‐C2 contains the HELICc domain, PIE1‐C5 contains the Coiled coil domain, and PIE1‐C6 contains the SANT domain (Figure S18a). Y2H assays revealed that PIE1‐C2 and PIE1‐C6 interact with AtD14 in the presence of *rac*‐GR24 (Figure S18b). We further investigated the interaction of yeast SWR1 with D14 and AtD14. The C‐terminal region of SWR1 (SWR1‐C) was divided into three fragments, with SWR1‐C2 containing the HELICc domain (Figure S19a). Y2H assays showed that SWR1‐C2 could interact with D14, but not with AtD14, in the presence of *rac*‐GR24 (Figure S19b). Functionally, overexpression of the *Arabidopsis PIE1* CDS in *ted1‐1* substantially rescued its mutant phenotype (Figure S20), whereas overexpression of yeast *SWR1* in *ted1‐1* resulted in only partial restoration (Figure 6d). Collectively, these results suggest that TED1 and its homologs have acquired a conserved role in SL signaling across plant species, potentially via the interaction between their C‐terminal domains and D14‐like proteins.

### 2.8 TED1‐Like Proteins Gain a Novel Binding Affinity for D53 via Their C‐Terminal Extension

Phylogenetic analysis showed that TED1 homologs are distributed across Fungi, Algae, Bryophyta, Pteridophyta, Gymnosperms, Dicots, and Monocots (Figure 6a). Notably, Gymnosperms were separated into two distinct clades: Cupressaceae species harbor SWR1‐like homologs with shorter C‐termini and display bushy plant types with absent or weak apical dominance, resembling the *ted1* mutants; in contrast, Taxaceae (*Taxus chinensis*) and Pinaceae (*Pinus koraiensis*) possess TED1‐like homologs with longer C‐termini and display tall tree crowns with strong apical dominance (Figure 6a).

Protein domain analysis showed that although the DEXDc and HELICc domains are conserved in the central region of TED1‐like proteins, the N‐terminal and C‐terminal domains vary among organisms, particularly between fungi, lower plants (Algae, Bryophyta, and Pteridophyta), and higher plants (Gymnosperms, Dicots and Monocots) (Figure 6a). Compared to lower Fungi and some Algae species, most land plants—and notably, even certain Algae—have evolved relatively longer C‐termini (Figure 6a). To explore the origin of this C‐terminal extension, we searched for homologous segments in the protein databases of Bacteria, Fungi, and Algae. Aside from SWR1‐like proteins in Algae, no homologous C‐terminal segments were identified (Figure S21), suggesting that the extended C‐terminus likely arose de novo in certain algal lineages. These findings imply that the C‐terminal extension of plant SWR1 homologs may provide the structural foundation to reinforce SL signaling.

Supporting this hypothesis, we found that the C2 fragment (containing the HELICc domain) of yeast SWR1 could interact with rice D14, but not with *Arabidopsis* AtD14, in the presence of SLs (Figures S18 and S19). In contrast, the longer C‐terminal regions of plant SWR1 homologs (such as TED1 and PIE1) contain additional segments (such as TED1‐C5 and C6; PIE1‐C6) that likely facilitate stable interaction between TED1's homologs and D14's homologs in an SL‐dependent manner (Figures S14, S18 and S19). Moreover, Co‐IP assays in tobacco revealed that yeast SWR1‐C did not interact with D53 (Figure S22a). However, expanded Co‐IP experiments showed that the extended C‐terminal regions from nine SWR1‐like proteins of nine different algal species all interacted with D53 (Figure S22b). This result is consistent with the robust interaction between TED1's C‐terminus and D53 observed in rice (Figure S15c), suggesting that TED1‐like proteins in some algal species have acquired the novel ability to bind D53‐like proteins through C‐terminal extension.

To genetically investigate the function of the extended C‐terminus, we fused the C‐terminus of PIE1 (SWR1N‐PIE1C, encompassing amino acids 1189–2055) and the C‐terminus of TED1 (SWR1N‐TED1C, encompassing amino acids 1193–2044) to the N‐terminus of yeast SWR1 (encompassing amino acids 1–1359), respectively. We then overexpressed these fusion genes in *ted1‐1* and compared the plant height and tiller number with those of the *ted1‐1* overexpressing the full‐length *SWR1* gene (Figure 6b). For each of the three transgenic constructs, two independent lines with comparable expression levels were selected (Figure 6c). The phenotypic analysis showed that, compared with the *ted1‐1* mutant, overexpression of *SWR1* slightly reduced the tiller number of *ted1‐1*. Compared with *SWR1* overexpression, overexpression of *SWR1N‐PIE1C* or *SWR1N‐TED1C* further decreased the tiller number of *ted1‐1*. Additionally, compared with *ted1‐1*, overexpression of *SWR1*, *SWR1N‐PIE1C*, or *SWR1N‐TED1C* all increased the plant height of *ted1‐1* (Figure 6d). Taken together, these findings indicate that the C‐terminal extension of TED1‐like proteins indeed can enhance their repression of rice tillering, which supports the hypothesis that the C‐terminal extension of plant SWR1 homologs may provide the structural foundation to reinforce SL signaling.

### 2.9 The TED1‐C5 Region May Serve as a Key Anchor for the Assembly of the TED1‐D14‐D53 Complex

To visualize the interactions of TED1 with D14 and D53, we employed AlphaFold3 to predict their putative interaction modes. The results showed that the three proteins can assemble into a compact ternary complex. The C‐terminal region of TED1 directly contacts both D53 and D14, which is consistent with our findings above (Figure S23a). At the interaction interface between TED1 and D14, multiple amino acid residues in the C5 segment of TED1 stabilize the binding of the two proteins via hydrogen bonds. Specifically, hydrogen bonds are formed between TED1^R1706^ and D14^S14^, TED1^H1697^ and D14^S11^, as well as TED1^Y1709^ and D14^Q58^. In addition, TED1^R1708^ simultaneously forms hydrogen bonds with D14^N110^ and D14^D112^, further enhancing the stability of the binding interface (Figures S23b and S9). Within the same region, TED1^S1698^ forms hydrogen bonds with D53^R113^ and D53^D153^, while TED1^R1706^ also interacts with D53^D153^ via hydrogen bonding. Notably, TED1^R1706^ also mediates a hydrogen bond with D14^S14^, indicating that this residue may play a key role in coordinating the assembly of the ternary complex (Figure S23c). These findings indicate that the TED1‐C5 region may play a key role in promoting the interaction among TED1, D14, and D53.

## 3 Discussion

**FIGURE 7:**
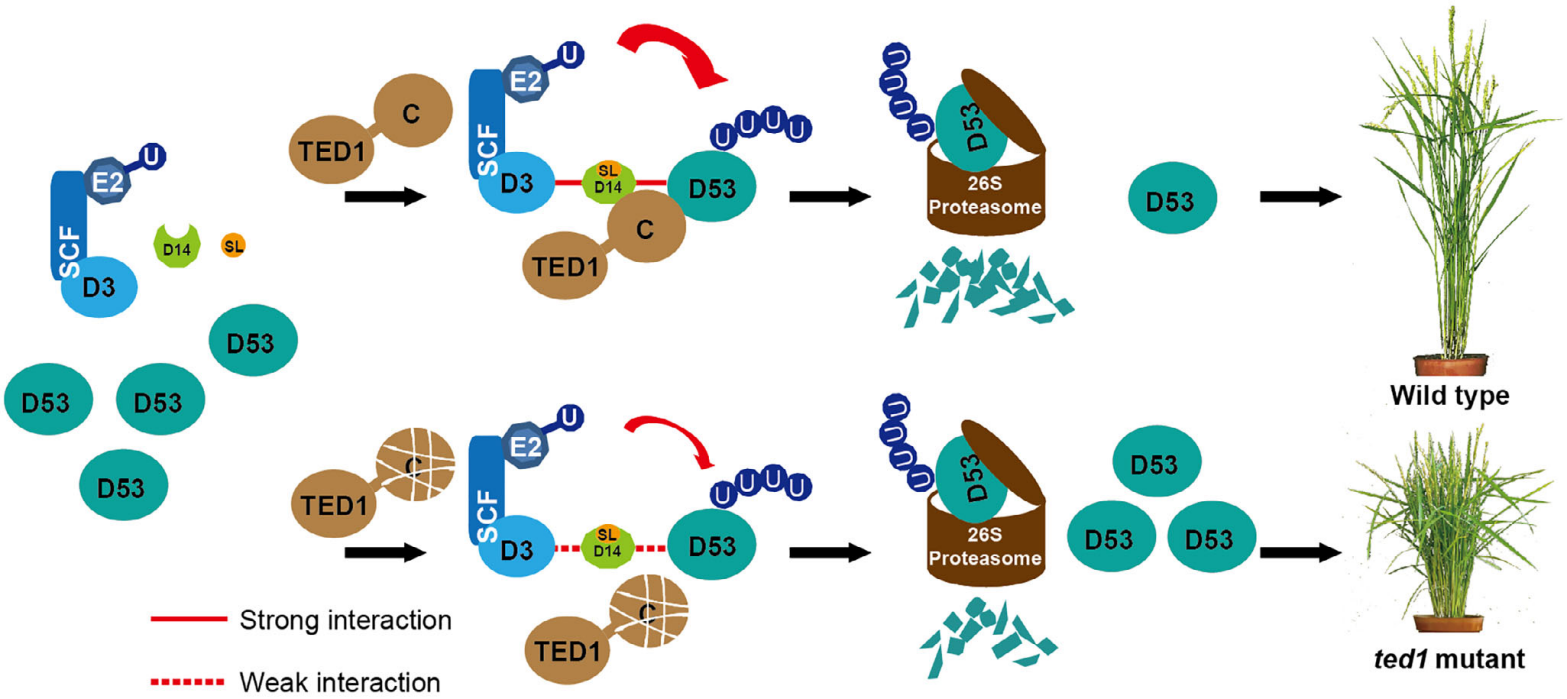
A schematic model for TED1's role in the SL signaling pathway. TED1 sustains a stable interaction between D3 and D14, as well as between D14 and D53 in the presence of SLs. This facilitates the efficient ubiquitination and degradation of the D53 protein, thereby suppressing rice tillering. Conversely, the C‐terminal destruction of TED1 results in a weakened interaction between D3 and D14, or between D14 and D53, even in the presence of SLs. This leads to the ineffective ubiquitination and degradation of the D53 protein, subsequently resulting in the accumulation of D53 protein, ultimately promoting rice tillering.

In this study, we identified TED1 as a novel regulator of SL signaling. First, five *TED1* mutant alleles (*ted1‐1* through *ted1‐5*) showed phenotypes characteristic of SL signaling mutants, including dwarfism and excessive tillering. Overexpression of *TED1* in the *ted1‐1* mutant effectively rescued these traits. Second, the expression of *TED1* is induced by Pi deficiency. Third, the *ted1‐1* mutant showed reduced sensitivity to SLs, whereas *ted1‐2* exhibited insensitivity to SLs, as evidenced by increased 4DO content in root exudates compared to WT. Fourth, TED1 physically interacted with D14 in the presence of SLs and with D53 independently of SLs. These interactions enhanced the association of D53 with D14 and of D14 with D3. Fifth, although the transcription levels of *D14* and *D3* genes are lower in *ted1‐1*, the protein abundance of D14 and D3 is unchanged in *ted1‐1*. Nevertheless, the ubiquitination of D53 is still significantly impaired in the *ted1‐1* mutant as in the *d14* mutant, thus leading to D53 accumulation, finally resulting in plant dwarfism and hyper‐tillering. Together, these findings demonstrate that TED1 facilitates the SL‐dependent assembly of the SCF^D3^‐D14‐D53 complex, thus promoting the ubiquitination and degradation of D53 to suppress rice tillering (Figure 7).

Notably, in SL‐signaling‐defective mutants (*d14*, *d3*, *ted1*, and *d53*) and SL‐biosynthesis‐defective mutants (*d27* and *d17*), *D10* showed relatively consistent transcriptional upregulation, indicating that *D10* may serve as a key target of negative feedback regulation [16, 48, 58]. In the SL biosynthesis pathway, *D10* encodes CCD8, which catalyzes the key rate‐limiting step of 9‐*cis*‐β‐10’‐carotene cleavage to carlactone (CL). This differential regulatory strategy is also conserved in *Arabidopsis* (*MAX4*), pea (*RMS1*), petunia (*DAD1*), and tomato (*SlCCD8*) [15, 59, 60], supporting the notion that the CCD8 (MAX4/RMS1/DAD1/D10) step represents an evolutionarily specialized regulatory node for SL signaling homeostasis.

Yeast SWR1, a homolog of TED1, functions as the core scaffold subunit of the SWR1 chromatin remodeling complex, recruiting partners such as ACTIN‐RELATED PROTEIN 6 (ARP6), ARP4, SWR1 COMPLEX SUBUNIT 2 (SWC2), SWC4, SWC6, YEAST ALL1‐FUSED GENE FROM CHROMOSOME 9a (YAF9A), and YAF9B to form the full complex [61, 62, 63, 64, 65]. SWR1 mediates chromatin remodeling by replacing H2A‐H2B histone dimers with H2A.Z‐H2B, a function conserved in humans (*Homo sapiens*) [42, 43, 61]. In *Arabidopsis*, PIE1 (a TED1 homolog) promotes the expression of the flowering repressor *FLC* via chromatin remodeling to repress flowering [54, 55, 56]. *PIE1* mutants exhibit pleiotropic phenotypes, including reduced plant height and increased branching [66]. Based on these roles, TED1 was initially hypothesized to regulate tillering through H2A.Z‐mediated chromatin remodeling. However, genetic analysis in rice revealed that the H2A.Z triple mutant displayed a tiller number similar to WT (Figure S6), paralleling observations in *Arabidopsis*, where the *hta9‐1/hta11‐2* double mutant exhibited only a slight increase in branch number [67]. Notably, this minor increase is similar to that observed in *h2av1*, *h2av1*/*h2av2*, and *h2av2*/*h2av3* rice mutants (Figure S6). Together, these results indicate that H2A.Zs play a limited role in regulating tillering and branching, supporting the idea that PIE1 and its homologs primarily control shoot branching in higher plants via the SL signaling pathway.

Consistent with this hypothesis, phylogenetic analysis showed that the C‐terminal extension of TED1 homologs originated de novo in ancestral algae [68, 69] before the emergence of Bryophyta (Figure 6a), coinciding with the appearance of D53‐like (SMXL) proteins [70]. This suggests that the C‐terminal extension may have co‐evolved with D53‐like proteins to facilitate their interaction. The C‐terminal extension is retained in TED1 homologs across most higher plants, except for species in the Cupressaceae family of Gymnosperms (Figure 6a). Biochemical analyses and three‐dimensional structure prediction support this notion: while the yeast SWR1 C2 domain (HELICc) interacts only with D14 but not AtD14 in the presence of SLs, plant TED1 and PIE1 proteins contain additional C‐terminal motifs (C5, C6) that enable stronger SL‐dependent interactions with D14‐like proteins and SL‐independent interactions with D53‐like proteins (Figure 4d; Figure S21a,b and Figures S12, S13, S15, S17, and S23).

In addition, these are distinct branching patterns among the three families in Gymnosperms: Pinaceae species exhibit very strong apical dominance, with first‐order branches arranged in regular whorls (3‐5 branches per whorl, mean of 4, in *Pinus koraiensis*), a species‐specific characteristic with limited variation [71, 72]; Taxaceae species display weaker apical dominance than pines, with irregularly alternate first‐order branches and an overall branching ratio of 8.57 in *Taxus yunnanensis* under full‐light conditions [73]; and Cupressaceae species show a “diffuse branching” pattern, in which each node of the main stem axis can produce 0, 1, or 2 first‐order lateral branches, and the extremely short internodes result in a very high density of first‐order branches per unit trunk length [74]. From a developmental perspective, these results showed that tree species harboring TED1‐like proteins with longer C‐termini tend to exhibit strong apical dominance (e.g., defined tree canopies), whereas Cupressaceae species with shorter C‐terminal SWR1‐like proteins display bushy forms with weak or absent apical dominance (Figure 6a) [75], a trait reminiscent of SL signaling mutants [3, 4, 23, 24, 25, 26, 27, 28, 29, 30, 31, 32, 33, 34, 35, 36, 37, 38, 39, 40, 41, 42, 43, 44]. Similarly, the *pie1* mutant showed significantly increased branches and reduced plant height [54, 55, 56]. Notably, overexpressing *Arabidopsis PIE1* with a longer C‐terminus substantially rescued the *ted1‐1* mutant phenotype (Figure S18). Further, we found that, compared to the overexpression of SWR1 alone in *ted1‐1*, yeast SWR1N fused with the C‐terminal regions of PIE1 and TED1 could further reduce the tiller number in *ted1‐1* (Figure 6d). These findings support the hypothesis that a new function can arise in an existing gene through C‐terminal extension, driving phenotypic evolution [76]. For *TED1*‐like genes, this extension appears to enhance apical dominance in higher plants by reinforcing SL signaling.

The core function of the SWR1 complex—the ATP‐dependent replacement of H2A with H2A.Z—is highly conserved across eukaryotes. Unlike yeast Swr1, which has a short C‐terminus interacting only with a few conserved subunits [62, 77], the extended C‐terminal regions of plant PIE1 and human SNF2‐RELATED CBP ACTIVATOR PROTEIN (SRCAP) have acquired multiple new protein interaction domains, recruiting species‐specific subunits not found in yeast, thereby diversifying their functions. In the plant SWR1 complex, the C‐terminal extension mediates interactions with plant‐specific subunits like CHROMATIN REMODELING 11/17(CHR11/17), METHYL‐CPG‐BINDING DOMAIN 9 (MBD9), and TUMOR NECROSIS FACTOR RECEPTOR‐ASSOCIATED FACTOR 1A/B (TRA1A/B), forming unique complex modules to regulate flowering time, photomorphogenesis, and stress responses [55, 78, 79, 80]. The animal SRCAP complex's C‐terminal extension recruits specific subunits (YL1, XPG), enabling it to regulate H2A.Z deposition and participate in DNA damage repair‐related chromatin remodeling, whereas the yeast SWR1 complex lacks these subunits and only indirectly affects genome stability via H2A.Z deposition [61, 62, 81, 82, 83]. Here, we showed that, besides their conserved roles in chromatin remodeling, the C‐terminal extension of TED1 enables it to interact with D14 and D53, thus facilitating the assembly of the SCF^D3^‐D14‐D53 complex to efficiently ubiquitinate the D53 protein, ultimately promoting its degradation to reinforce SL signaling (Figure 7). This neo‐functionalization likely represents a new mechanism of gene evolution distinct from previously reported models [84, 85].

## 4 Conclusion

This study identifies TED1, a scaffold subunit of the SWR1‐like chromatin remodeling complex in rice, as a positive regulator of SL signaling. Biochemical and genetic evidence, along with three‐dimensional structure prediction, demonstrate that TED1 interacts with D53 independently of SLs and with the receptor D14 in an SL‐dependent manner through its C‐terminal domain. This enhances the D14‐D53 and D14‐D3 interactions, promotes the assembly of the SCF^D3^‐D14‐D53 complex for the efficient ubiquitination and degradation of D53, and finally suppresses rice tillering. Furthermore, this study reveals that the C‐terminal extension of TED1‐like proteins has acquired a novel function during evolution—reinforcing SL signaling. In summary, this study not only elucidates the underlying new molecular mechanism of TED1‐mediated SL signaling regulation, but also provides new insights into the functional evolution of chromatin remodeling factors in plant hormone signal transduction.

## 5 Materials and Methods

### 5.1 Plant Materials and Growth Conditions

The photoperiod‐insensitive rice cultivar Kitaake (*Oryza sativa* L. subspecies *japonica*) served as the WT in this study. The plants were grown in the field at two locations: Beijing (40°13’N, 116°13’E) and Sanya (18°48’N, 110°02’E), or cultivated in an incubation chamber (Ruihua, HP1500GS) under controlled conditions of 12 h of light at 30°C and 12 h of darkness at 25°C, with 70% relative humidity and a light intensity of 800 µmol m^−2^s^−1^.

### 5.2 Vector Construction and Genetic Transformation

To knock out *TED1*, a CRISPR/Cas9 vector targeting *TED1* was constructed. The target sites were designed using CRISPR‐P v2.0 (hzau.edu.cn) and the *RNAfold* WebServer (univie.ac.at). The CRISPR/Cas9 vector for *TED1* was introduced into Kitaake through *Agrobacterium tumefaciens*‐mediated transformation. Mutants of *d3*, *d14*, *d27*, *d17*, and *d10* used in this study were generated using CRISPR/Cas9 technology in the Kitaake genetic background. Additionally, the *h2av1*/*h2av2*/*h2av3* triple mutant and *d53* knockout in the *ted1‐1* background were also created using the same approach. The sgRNA sequences were listed in Table S2.

For the construction of *pActin::TED1*, the full‐length coding sequence (CDS) of *TED1* was amplified with the primers in Table S3 and cloned into pCAMBIA2300 and pCAMBIA1300‐3×Flag vectors, respectively. These vectors were then sequenced for their accuracy after being transformed into *E*. coli *Trans*Stbl3 competent cells (TransGen Biotech). *pActin1::TED1* was introduced into *ted1‐1* via *Agrobacterium tumefaciens*‐mediated transformation.

To create the *pActin1::D14‐3*×*Flag* vector, the full‐length coding sequence of *D14* (excluding the termination codon) was amplified using the primers listed in Table S3 and cloned into the pCAMBIA2300‐3×Flag vector. *pActin1::D14‐3*×*Flag* was introduced into *ted1‐1* through *Agrobacterium tumefaciens*‐mediated transformation.

The *pActin1::PIE1* vector was constructed by amplifying the full‐length coding sequence of *PIE1* with the primers provided in Table S3 and cloning it into the pCAMBIA2300 vector. This vector was then introduced into *ted1‐1* using *Agrobacterium tumefaciens*‐mediated transformation.

Similarly, the *pActin1::SWR1* vector was created by amplifying the full‐length coding sequence of *SWR1* with the primers in Table S3 and cloning it into the pCAMBIA2300 vector. Subsequently, *pActin1::SWR1* was introduced into *ted1‐1* via *Agrobacterium tumefaciens*‐mediated transformation.

### 5.3 RNA Extraction, RT‐PCR, and RT‐qPCR

Various plant tissues were harvested and stored in liquid nitrogen or at ‐80°C refrigerator. Total RNA extraction was performed using the ZR Plant RNA MiniPrep Kit (Zymo). Reverse transcription‐PCR (RT‐PCR) was carried out using the QuantiTect Reverse Transcription Kit (Qiagen). For real‐time quantitative PCR (RT‐qPCR), SYBR Premix ExTaq (Takara) and an ABI7500 Real‐Time PCR System were utilized, with the rice ubiquitin gene serving as the internal control. The average expression levels and standard deviations were calculated from three experimental replicates. The RT‐qPCR primers used in the experiment were listed in Table S4.

### 5.4 Functional Domain Prediction and Protein Sequence Alignment

The TED1 protein sequence underwent functional domain analysis using SMART (SMART: Main page (embl‐heidelberg.de)) and a homolog search using BLASTp from NCBI (Protein BLAST: search protein databases using a protein query (nih.gov)). Protein sequence alignment and plotting were conducted using ClustalX and BioEdit, respectively.

### 5.5 Subcellular Localization

The *TED1* codon‐optimized CDS sequence was amplified using the primers from Table S3 and cloned into the pAN580‐GFP vector. The nucleus marker D53‐mCherry was utilized, and both constructs were co‐transformed into rice protoplasts. A laser scanning confocal microscope (LSM 700; Carl Zeiss) was used to observe fluorescence signals.

A similar approach was followed for cloning the TED1 codon‐optimized CDS sequence into the pCAMBIA1305‐GFP vector, which was then transformed into tobacco (*N. benthamiana*) leaves through *Agrobacterium tumefaciens*. The tobacco leaves were observed for fluorescence signals using a laser scanning confocal microscope.

### 5.6 Treatment With 5DS and *rac*‐GR24

The responses of WT, *ted1‐1*, *ted1‐2*, *d24*, and *d27* to SLs were studied by hydroponically germinating seeds for 1 week, followed by 6 weeks of growth in nutrient solution with or without 1 µM 5DS or *rac*‐GR24. Plant phenotypes were observed and analyzed. 5DS ((±)‐5‐deoxystrigol, CX96323‐5MG, Chiralix), *rac*‐GR24 (41012ES25, YEASEN).

### 5.7 The Quantification of SLs

The extraction and quantification of strigolactones (SLs) from root exudates were carried out following the methods outlined in Zhou et al. (2013), Jiang et al. (2013), and Chu et al. (2017) with some modifications. Root exudates were collected after approximately 2 weeks of growth in a phosphorus‐deficient medium. Subsequently, D_3_‐*ent*‐2’‐*epi‐*5DS (D_3_‐4DO) was introduced as an internal standard for quantification. For the root exudate, purification of SLs was achieved using an Oasis HLB cartridge. The final purified samples were eluted with acetone and re‐dissolved in acetonitrile.

The analysis of SLs was conducted using an UPLC‐MS/MS system comprising an UPLC system (Waters) equipped with a BEH C18 column (1.7 µM, 100 × 2.1 mm, Waters) and a QTRAP 6500 mass spectrometer (AB SCIEX) equipped with an ESI source. The gradient commenced from 50% mobile phase A (0.05% acetic acid in water) and increased mobile phase B (0.05% acetic acid in acetonitrile) from 30% to 90% in 5 min at 25°C, with a flow rate of 0.3 mL/min. The MRM mode was used for quantification, and the selected MRM transitions were 331.2 > 216.1 for *ent*‐2’‐*epi*‐5DS (4DO) and 337.2 > 221.1 for D_3_‐4DO. The analyses were performed in three biological replicates.

### 5.8 Yeast Two‐Hybrid (Y2H) Assay

The Y2H assay protocol followed the yeast transformation manual from Clontech (Yeastmaker Yeast Transformation System 2 User Manual). For the experimental setup, different coding sequences were amplified using the primers listed in Table S5 and cloned into pGBKT7 or pGADT7 vectors, respectively. pGBKT7, containing a GAL4 DNA‐binding domain, served as the “Bait”, while pGADT7, containing a GAL4 DNA‐activation domain, functioned as the “Prey”. These vectors were co‐transformed into the yeast strain AH109 (Clontech) and cultivated on SD/‐Leu‐Trp selection medium for 3 days at 30°C. Subsequently, SD/‐Leu‐Trp‐His‐Ade selection medium with or without *rac*‐GR24 and SD/‐Leu‐Trp selection medium were employed to assess the interactions.

### 5.9 Bimolecular Fluorescence Complementation (BiFC)

The *TED1*, *D14*, *D53*, and *D3* coding sequences were amplified using the primers from Table S3 and cloned into the P2YN and P2YC vectors, respectively. These vectors were then transformed into the *Agrobacterium tumefaciens* strain EHA105. According to the experimental design, the EHA105 carrying the different vectors was infiltrated into tobacco (*N. benthamia*) leaves. Laser scanning confocal microscopy was utilized to visualize the green fluorescent protein.

### 5.10 Co‐Immunoprecipitation (Co‐IP)

The *TED1‐C* and *D14* coding sequences were amplified using the primers listed in Table S3 and then cloned into the binary vectors pCAMBIA1300 and pCAMBIA1305, respectively, to generate *TED1‐C‐3×Flag* and *GFP‐D14* vectors. *Agrobacterium* was used to infect tobacco leaves for co‐expressing the two proteins, following the description previously. After two days, 10 µM *rac*‐GR24 and 10 µM MG132 were injected into tobacco leaves, which were then collected the following day.

NB1 buffer containing 50 mM Tris‐MES (pH 8.0), 0.5 M Sucrose, 1 mM MgCl_2_, 10 mM EDTA (pH 8.0), 5 mM DTT, Protease Inhibitor Cocktail Tablets (1 pill per 25 mL NB1 buffer, Roche), and 50 µM MG132 (Calbiochem) was utilized for protein extraction from the tobacco leaves. The leaves were ground and weighed, and 1 g samples were incubated in 2.5 mL NB1 buffer at 4°C for 30 min. Plant residues were removed by centrifugation at 12,000 rpm for 5 min at 4°C. Protein G was employed to eliminate non‐specific hybrid proteins. Anti‐DDDDK(FLAG)‐tag mAb‐Magnetic Beads (MBL) were washed twice with NB1 buffer. Subsequently, 20 µL beads were added for every 1 mL of protein extract and incubated at 4°C for 4 h. GFP antibody (Anti‐GFP pAb‐HRP‐DirecT, MBL) and Flag antibody (Anti‐DDDDK‐tag mAb‐HRP‐DirecT, MBL) were used to detect GFP‐D14 and TED1‐C‐3×Flag protein, respectively.

### 5.11 In Vitro Ubiquitination

The *D53* CDS sequence was amplified using the primers from Table S3 and cloned into the pGEX‐4T‐1 vector (GE Healthcare) for prokaryotic expression of the GST‐D53‐His protein. GST magnetic beads (BeaverBeads GSH) were used to capture the GST‐D53‐His protein, but elution was not performed. WT, *ted1‐1*, and *d14* rice plants were hydroponically cultivated for 3 weeks, and 0.5 cm samples were taken from the basal part of the rice stem. TNM buffer (25 mM Tris‐HCl (pH 7.5), 10 mM NaCl, 10 mM MgCl_2_, 40 µM MG132, 5 mM DTT (dithiothreitol), Protease Inhibitor Cocktail Tablets (1 pill per 50 mL TNM buffer), 10 mM ATP) was used to extract the total proteins of these samples. One‐gram samples were incubated in 2 mL TNM buffer on ice for 15 min. Plant residues were removed by centrifugation at 13,000 rpm for 10 min at 4°C. Magnetic beads‐GST‐D53‐His complexes were incubated with rice total proteins at room temperature for 15 min. GST antibody (Anti‐GST‐tag pAb‐HRP‐DirecT, MBL) and ubiquitin antibody (CST, Ubiquitin (P4D1) Mouse mAb #3936) were used to detect the GST‐D53‐His and GST‐D53‐His(Ubi) shifted bands, respectively.

### 5.12 In Vivo Degradation Experiment of Protein

The WT, *ted1‐1*, and *d14* plants were hydroponically cultivated for 2 weeks. The seedlings were submerged in water for 3 h to promote the In vivo accumulation of the D53 protein. Subsequently, the plants were treated with 1 mM cycloheximide (CHX, INALCO, 1758–9301) for 15 min. Half of the seedlings were sampled as the control group, while the remaining seedlings were treated with 5 µM *rac*‐GR24 for 2 h. The samples were then ground, and total proteins were extracted using a PBS protein extract buffer (0.2 µL Staurosporine (1 mM, Cell Signaling Technology), 10 µL MG132 (10 mM, Calbiochem), 10 µL Protease Inhibitor Cocktail (100×, Sigma), Protease Inhibitor Cocktail Tablets (1 pill per 50 mL PBS buffer, Roche), 10 µL PhosSTOP (1 pill per 50 mL NB1 buffer, Roche), 200 µL 5× protein loading buffer, diluted with PBS buffer to 1 mL). The D53 antibody and the housekeeping protein HSP82 (Beijing Protein Innovation Co., Ltd) were utilized to detect the target bands, respectively.

### 5.13 Semi‐In Vivo Pull‐DOWN Analysis

The D14 CDS sequence was amplified using the primers from Table S3 and cloned into the pET‐28a vector to express the His‐D14 protein in *E. coli* cells. The His‐D14 protein was purified using His‐tag magnetic beads (BeaverBeads IDA‐Nickel) and eluted with a 400 mM imidazole elution solution. The WT and *ted1‐1* seedlings were hydroponically cultivated for 4 weeks, and samples were taken from the basal part of the rice stem. The samples were ground, and total proteins were extracted using RIPA buffer (comprising 0.2 µL Staurosporine (1 mM, Cell Signaling Technology), 10 µL MG132 (10 mM, Calbiochem), 10 µL Protease Inhibitor Cocktail (100×, Sigma), Protease Inhibitor Cocktail Tablets (1 pill per 50 mL RIPA buffer, Roche), PhosSTOP (1 pill per 50 mL RIPA buffer, Roche), 2 µL TritonX‐100, diluted with RIPA buffer to 1 mL). His‐tag magnetic beads were utilized to collect purified His‐D14 proteins, which were then incubated with total proteins on ice for 30 min. Experimental groups were treated with 50 µM *rac*‐GR24, while the control groups were treated with DMSO (Dimethyl sulfoxide). D53, D3, and His antibodies were employed to detect the target bands.

### 5.14 Phosphate Starvation Treatment Experiment

The treatment method was adapted from Haider et al. (2023) with some modifications [57]. Seeds of the wild‐type rice cultivar Kitaake (*Oryza sativa* L.) were surface‐sterilized and soaked in the dark at 28°C for 2 days to induce germination. Germinated seedlings were then transferred to a phosphate‐sufficient (+P) Yoshida rice nutrient solution (Coolaber, NSP1040) and pre‐cultured for 7 days. Plants were cultivated in an incubation chamber (Ruihua, HP1500GS) under controlled conditions of 12 h light at 30°C and 12 h darkness at 25°C, with 70% relative humidity and a light intensity of 800 µmol m^−^^2^ s^−^^1^. The nutrient solution was renewed every 3 days. After 7 days of pre‐culture, seedlings were transferred to either the phosphate‐sufficient control group, which was maintained in a +P nutrient solution, or the phosphate‐deficient treatment group (Coolaber, NSP1040‐P), and treated for 1, 3, 7, and 8 days, respectively. Root samples from both control and treatment groups were harvested at 1, 3, 7, and 8 days after treatment, immediately frozen in liquid nitrogen, and stored at ‐80°C for subsequent RNA extraction and gene expression analysis.

### 5.15 Pull‐Down Assay

The *TED1* codon‐optimized CDS sequence was amplified using the primers provided in Table S3 and cloned into the pET28a vector to express the His‐TED1 protein in *E. coli* cells. *D14*, *D53*, and *D3* coding sequences were amplified and cloned into the pGEX‐4T‐1 vector. Magnetic beads‐His‐TED1 were incubated with GST, D14, D53, and D3 proteins at 4°C for 30 min in 600 µL binding buffer (comprising 20 mM Tris‐HCl, pH 7.6, 0.1 M NaCl, 2.5 mM β‐ME). Subsequently, the beads were washed three times with a washing buffer (20 mM Tris‐HCl, pH 7.5, 500 mM NaCl, 0.5% Triton X‐100). Following the washes, GST antibody (Anti‐GST‐tag pAb‐HRP‐DirecT, MBL) and His antibody were used to detect the target bands.

### 5.16 Antibody Production and Endogenous Protein Detection

Antibodies against D14 and D3 were custom‐produced by Shanghai Youlong Biotechnology Co., Ltd. The immunogen regions used for antibody production were amino acids 60–317 for D14 and amino acids 255–448 for D3. The D53 antibody was the same peptide as that used in Zhou et al., 2023 (Nature) [41]. Total proteins were extracted from the basal stems of rice using the PBS protein extraction buffer described in Section 5.12. Protein samples were boiled according to the following procedure: 80°C for 5 min, followed by 95°C for 10 min. The D14, D3, and D53 antibodies were used at a dilution of 1:1,000 and incubated for at least 3 h.

### 5.17 Statistical Analysis

Data are presented as the mean ± standard deviation (SD). Sample sizes (*n*) and the number of experimental replicates are provided in the corresponding figure legends. Statistical significance between two groups was determined using a two‐tailed Student's t‐test. For comparisons involving more than two groups, one‐way or two‐way analysis of variance (ANOVA) was performed, followed by Tukey's post‐hoc test for multiple comparisons, as indicated in the figure legends. The chi‐square test was used to analyze the segregation ratio in genetic crossing populations. The threshold for statistical significance was set at p < 0.05, with the following levels indicated: *p* < 0.05, *; *p* < 0.01, **. All statistical analyses were performed using SPSS version 22.0.

## Author Contributions

J.W., Q.L., Z.C., and C.W. supervised the study and revised the manuscript. S.L., M.F., and W.L. performed the experiments. S.L. wrote the manuscript. R.M., J.Q., F.W., C.Z., B.L., J.S., and Z.X. assisted with the experiments and provided experimental methods. Z.L. and H.W. advised on the initial research direction. Y.X. provided guidance on protein expression and predicted and analyzed the three‐dimensional structures of protein–protein interactions. Z.Z., C.L., S.Z., Y.R., X.W., and X.G. provided technical platforms and methodological support for the experiments.

## Funding

The National Key Research and Development Program of China (2022YFF1002903, 2022YFD1200104), Innovation Program of Chinese Academy of Agricultural Sciences (CAAS‐CSCB‐202401), Inner Mongolia Innovation Center of Biological Breeding Technology (2026), Central Public‐interest Scientific Institution Basal Research Fund (No. Y2024YJ13), and the National Natural Science Foundation of China (31671769).

## Conflicts of Interest

The authors declare no conflicts of interest.

## Supporting information




**Supporting File**: advs77062‐sup‐0001‐SuppMat.docx.

## Data Availability

The materials that support the findings of this study are available from the corresponding author upon reasonable request.
