# Supplementary material for: TED1 Promotes the Assembly of SCFD3‐D14‐D53 to Reinforce Strigolactone Signaling in Rice
Source: Adv Sci (Weinh). 2026 Sep 9:e77062. Online ahead of print. doi: 10.1002/advs.77062 (PMC13556982; doi:10.1002/advs.77062)
Supplement: Supplementary file 1 [file ADVS-9999-e77062-s001.docx]

Supporting Information

**TED1 promotes the assembly of SCF^D3^-D14-D53 to reinforce strigolactone signaling in rice**

*Sheng Luo, Chuanyin Wu, Miao Feng, Wenfan Luo, Rong Miao, Jinsheng Qian, Fan Wang, Chunlei Zhou, Bojuan Liu, Jiale Shao, Ziqi Xun, Yehui Xiong, Zhichao Zhao, Cailin Lei, Shanshan Zhu, Yulong Ren, Xin Wang, Xiuping Guo, Haiyang Wang, Zichao Li, Zhijun Cheng^*^, Qibing Lin^*^, and Jianmin Wan^*^*

**Figure S1. *TED1* gene mutants generated by CRISPR/Cas9 technology.**

(**a**) Phenotype of the four *TED1* allelic mutants. The wild type (WT) is Kitaake. Scale bar: 15 cm. (**b**) Mutated sequences of the *TED1* gene in five *ted1* mutants. Mutations were generated at two different target sites. sgRNA, small guide RNA, are indicated by blue lines. PAMs, protospacer adjacent motifs, are highlighted in red letters. (**c**) Plant height and tiller number of WT and the five *TED1* mutants. The asterisks indicate the significance of the differences between the mutants and the wild type. Values are means ± s.d. (*n* > 10). *P*-values are from a two-sided Student’s *t*-test (*, *P* < 0.05; **, *P* < 0.01).

**Figure S2. The mutant phenotype of *ted1-1* is controlled by a single recessive nuclear gene.**

(**a**) F_1_ plants from reciprocal crosses between WT and *ted1-1*. Scale bar: 15 cm. (**b**) Plant height and tiller number of WT, *ted1-1*, and their F_1_ plants. Values are means ± s.d. (*n* > 13). *P*-values are from a one-way ANOVA test with Tukey’s correction (significant differences between different letters). (**c**) Segregation of the *ted1-1* phenotype in F_2_ populations derived from the reciprocal crosses. *P*-values are from the chi-square test.

**Figure S3. Visual comparison of internode length between WT and *ted1-1.***

P, panicle; I, first internode; II, second internode; III, third internode; IV, fourth internode; V, fifth internode. White arrows indicate stem nodes. Scale bar: 10 cm.

**Figure S4. Multiple protein sequence alignment among TED1 and its homologs.**

TED1 protein was predicted to have three main domains: HSA (marked by a deep blue box), DEXDc (marked by red boxes), HELICc (marked by orange boxes), and two Coiled coil (marked by light blue boxes) domains by SMART ([embl-heidelberg.de)](http://smart.embl-heidelberg.de/). The domain names were obtained from the SMART website. Sequence alignment was performed between TED1 (*Oryza sativa*, Os02g0689800), PIE1 (*Arabidopsis thaliana*, AT3G12810), and SWR1 (*Saccharomyces cerevisiae*, NCBI gene ID: 851934).

**Figure S5. The interaction between** **TED1 and H2A.Zs in rice.**

(**a**) The TED1 protein was divided into three parts according to its domains: TED1-N (TED1 N-terminal, 1-500 AA), TED1-M (TED1 middle, 240-1010 AA), and TED1-C (TED1 C-terminal, 986-2044 AA). AA, amino acid. (**b**) H2AV1, H2AV2, and H2AV3 were found to interact with TED1-N only. AD, activation domain; BD, binding domain; -LT, SD -Leu/-Trp; -LTH, SD -Leu/-Trp/-His; -LTHA, SD -Leu/-Trp/-His/-Ade.

**Figure S6. Phenotypes of CRISPR/Cas9 knockout mutants related to rice H2A.Zs.**

(**a**) Phenotype of *ted1-1*, *h2av1*, *h2av2*, and *h2av3* single mutants, *h2av1*/*h2av2*, *h2av1*/*h2av3*, and *h2av2*/*h2av3* double mutants*,* and *h2av1*/*h2av2*/*h2av3* triple mutant. (**b**) Comparison of plant height and tiller number of WT, *ted1-1*, *h2av1*, *h2av2*, *h2av3*, *h2av1*/*h2av2*, *h2av1*/*h2av3*, *h2av2*/*h2av3*, and *h2av1*/*h2av2*/*h2av3*. The asterisks indicate the significance of the difference between the mutants and the wild-type Kitaake. Scale bars: 15 cm. Values are means ± s.d. (*n* > 15). *P*-values are from two-sided Student’s *t*-tests (*, *P* < 0.05; **, *P* < 0.01).

 **Figure S7. Examination of transcriptional relationships between *TED1* and strigolactone pathway genes.**

(**a**) Expression levels of the strigolactone pathway genes *D53*, *D3*, *D14*, *D27*, *D17*, *D10*, and *FC1* in WT and *ted1-1*. Values are means ± s.d. (*n* = 3). *P*-values are from two-sided Student’s *t*-tests (*, *P* < 0.05; **, *P* < 0.01). (**b**) Expression level of *TED1* in *d3*, *d14*, *d27*, *d17*, *d10*, and *d53* mutants, as well as in their corresponding WTs. The *d3*, *d14*, *d27*, *d17*, and *d10* mutants were generated using CRISPR/Cas9 technology in the genetic background of Kitaake. The *d53* mutant is a dominant mutant in the genetic background of NL8 (Nonglin 8). The asterisks indicate the significance of the difference between the mutants and the wild type. Values are means ± s.d. (*n* = 3). *P*-values are from a two-sided Student’s *t*-test (*, *P* < 0.05; **, *P* < 0.01).

**Figure S8. Effects of phosphate starvation treatment on *TED1* expression.**

Relative expression levels of *TED1* in wild-type rice seedlings grown under phosphate-sufficient and phosphate-starvation conditions for the indicated number of days. Values are means ± s.d. (*n* = 3). *P*-values are from two-sided Student’s *t*-tests (*, *P* < 0.05; **, *P* < 0.01).

**Figure S9. Quantification of 4DO and ORO in root exudates of WT, *ted1-1* and *ted1-2.***

(**a**) Comparison of root exudates 4DO between WT and *ted1-1* by Liquid chromatography-tandem mass spectrometry (LC-MS/MS). ng/g FW, ng per gram fresh weight. Values are means ± s.d. (*n* = 3). *P*-values are from two-sided Student’s *t*-tests (**, *P* < 0.01). (**b**) Orobanchol (ORO) content in WT, *ted1-1*, and *ted1-2* plants was not detected (ND).

**Figure S10. Overexpressing *D14* in *ted1-1* did not restore its phenotype to that of the WT.**

(**a**) Phenotype of WT, *ted1-1*, and the three independent transgenic lines overexpressing *D14* in the *ted1-1* mutant background. Scale bar: 15 cm. (**b**) Expression level of *D14* in WT, *ted1-1*, and the transgenic lines overexpressing *D14* in the *ted1-1* mutant background. Values are means ± s.d. (*n* = 3). *P*-values are from one-way ANOVA tests with Tukey’s correction (significant differences between different letters). (**c**) Plant height and tiller number of WT, *ted1-1*, and the transgenic lines overexpressing *D14* in the *ted1-1* mutant. Values are means ± s.d. (*n* > 13). *P*-values are from one-way ANOVA tests with Tukey’s correction (significant differences between different letters).

**Figure S11. The protein level detection of D3 and D14*.***

(**a**) The protein levels of D3 and D14 were comparable between WT and the *ted1-1* mutant. Numbers represent the protein band intensity of D3 and D14 relative to the internal reference protein HSP82. Protein content was determined in the basal stems of 6-week-old seedlings. (**b**, **c**) Three independent replicate experiments were performed for the detection of D3 and D14 protein levels in WT and *ted1-1*. R1, R2, and R3 represent Repeat 1 (**a**), Repeat 2, and Repeat 3, respectively. (**d**) The protein levels of D14 in WT, *ted1-1*, and three independent *D14* overexpression lines in the *ted1-1* background (L1, L3 and L4) were compared. Numbers represent the relative band intensity of D14 normalized to the internal reference protein HSP82. Protein content was determined in the basal stems of 2-week-old seedlings.

**Figure S12. *rac*-GR24 promotes the interaction between TED1 and D14.**

Pull-down assays examining the interactions between TED1 and D14, D53, and D3 were performed in three independent replicates. The relative band intensities of the GST-D14 bands after IP were quantified. The relative band intensity of GST-D14 was calculated by normalizing the intensity of GST-D14 after IP to that of His-TED1 after IP. R1, R2, and R3 represent Repeat 1 (**Figure 4D**), Repeat 2, and Repeat 3, respectively.

**Figure S13. The interaction between TED1-C and D14 was dependent on *rac*-GR24.**

(**a**) Yeast two-hybrid assay demonstrated the interaction between TED1 (TED1-N, TED1-M, and TED1-C) and D14 in culture medium with or without *rac*-GR24. AD, activation domain; BD, binding domain; -LT, SD -Leu/-Trp; -LTHA, SD -Leu/-Trp/-His/-Ade. (**b**) *In vivo* Co-IP analysis demonstrates the interaction between the TED1-C-FLAG fusion protein and the GFP-D14 fusion protein in the leaves of *N. benthamiana*.

**Figure S14. Identification of domains in the TED1 C-terminal that interact with D14.**

(**a**) TED1-C was segmented into six parts based on potentially functional domains: C1 (986-1108 AA), C2 (1109-1192 AA), C3 (1193-1420 AA), C4 (1421-1521 AA), C5 (1522-1807 AA), and C6 (1808-2044 AA). AA, amino acid. (**b**) In the presence of 50 µM *rac*-GR24, C2, C5 and C6 were found to interact with D14. AD, activation domain; BD, binding domain; -LT, SD -Leu/-Trp; -LTHA, SD -Leu/-Trp/-His/-Ade.

**Figure S15. Examining the interaction of TED1-NM or TED1-C with D53 or D3 using *in vivo* Co-IP assays.**

(**a**) TED1 was segmented into two parts: TED1-NM (TED1 N-terminal and middle, 1-1010 AA) and TED1-C (TED1 C-terminal, 986-2044 AA). AA, amino acid. (**b**) *In vivo* Co-IP analysis revealed that the TED1-NM-FLAG fusion protein did not interact with the D53-GFP fusion protein in the leaves of *N. benthamiana*. (**c**) *In vivo* Co-IP analysis showed the interaction between the TED1-C-FLAG fusion protein and the D53-GFP fusion protein in the leaves of *N. benthamiana*. (**d**, **e**) *In vivo* Co-IP analysis showed that neither TED1-NM-FLAG (**d**) nor TED1-C-FLAG (**e**) interacted with the D3-GFP fusion protein in the leaves of *N. benthamiana*.

**Figure S16. Amino acid sequence alignment of TED1-C-MT1 and TED1-C-MT2 and their interaction analysis with components of the SL signaling pathway.**

(**a**) Amino acid sequence alignment of TED1-C-MT1 and TED1-C-MT2. The protein sequences of both TED1-C-MT1 and TED1-C-MT2 start from the C1 fragment (**Figure S15a**). Black shading indicates identical amino acid residues. (**b**) Protein interactions of TED1-C-MT1 and TED1-C-MT2 with D14, D53, and D3 detected by the yeast two-hybrid assay. Both TED1-C-MT1 and TED1-C-MT2 can interact with D14 in a *rac*-GR24-dependent manner, whereas no obvious interaction was observed with D53 or D3. AD, activation domain; BD, binding domain; -LT, SD -Leu/-Trp; -LTHA, SD -Leu/-Trp/-His/-Ade.

**Figure S17. Quantification of relative protein band intensities from three independent replicates of the protein assays shown in Figure 5.**

(**a**) Relative band intensity of D53 normalized to the internal reference protein HSP82 in WT, *ted1-1*, and *d14*. R1, R2, and R3 represent Repeat 1 (**Figure 5a**), Repeat 2, and Repeat 3, respectively. (**b**) Relative band intensity of D53 normalized to the internal reference protein HSP82 in the degradation assay. R1 (**Figure 5b**). (**c**) Relative band intensity of D53 after IP normalized to His-D14 after IP in the in vitro pull-down assay. R1 (**Figure 5c**). (**d**) Relative band intensity of D3 after IP normalized to His-D14 after IP in the in vitro pull-down assay. R1 (**Figure 5e**). (**e**) Relative band intensity of D14-FLAG after IP normalized to GFP-D53 after IP in the *Nicotiana benthamiana* leaf Co-IP assay. R1 (**Figure 5f**). (**f**) Relative band intensity of D14-FLAG after IP normalized to GFP-D3 after IP in the *Nicotiana benthamiana* leaf Co-IP assay. R1 (**Figure 5g**).

**Figure S18. Investigating the interaction between PIE1-C segments and AtD14 via a yeast two-hybrid assay.**

(**a**) The PIE1-C (C-terminal of PIE1) was segmented into five parts based on functional domains: C1 (982-1104 AA), C2 (1105-1188 AA), C34 (1189-1295 AA), C5 (1296-1669 AA), and C6 (1670-2055 AA). AA, amino acid. (**b**) In the presence of 50 µM *rac*-GR24, C2 and C6 were found to interact with AtD14. DMSO was added as a control group. AD, activation domain; BD, binding domain; -LT, SD -Leu/-Trp; -LTHA, SD -Leu/-Trp/-His/-Ade.

**Figure S19. The yeast two-hybrid assay showed potential interaction sites of yeast SWR1-C that interact with rice D14 but not with Arabidopsis AtD14**.

(**a**) SWR1-C (C-terminal of SWR1) was segmented into three parts based on functional domains: C1 (1,153-1,275 AA), C2 (1,276-1,359 AA), and C3 (1,360-1,514 AA). AA, amino acid. (**b**) In the presence of 50 µM *rac*-GR24, C2 was found to interact with D14. DMSO was added as a control. AD, activation domain; BD, binding domain; -LT, SD -Leu/-Trp; -LTHA, SD -Leu/-Trp/-His/-Ade.

**Figure S20. Overexpression of Arabidopsis *PIE1* significantly restored the mutant phenotype of *ted1-1.***

(**a**) Phenotypic comparison between WT, *ted1-1*, and a *PIE1*-overexpressing transgenic line in *ted1-1*. Scale bar: 15 cm. (**b**) Expression level of *PIE1* in WT, *ted1-1*, and a *PIE1*-overexpressing transgenic line in *ted1-1*. Values are means ± s.d. (*n* = 3). *P*-values are from one-way ANOVA tests with Tukey’s correction (significant differences between different letters). (**c**) Plant height and tiller number of WT, *ted1-1*, and a *PIE1*-overexpressing transgenic line in *ted1-1*. Values are means ± s.d. (*n* > 15). *P*-values are from one-way ANOVA tests with Tukey’s correction (significant differences between different letters).

**Figure S21. A DELTA-BLAST search with TED1's C-terminal protein sequence revealed high conservation of the C2 (HELICc domain) in different species.**

The search criteria for DELTA-BLAST in NCBI were respectively restricted to Bacteria (**a**), Fungi (**b**), and Algae (**c**). The C2 (HELICc domain) was highly conserved in almost all species.

**Figure S22. Examination of the interaction between D53 in rice and the C-termini of SWR1-like proteins from Algae.**

(**a**) *In vivo* Co-IP analysis showed no interaction between the SWR1-C-FLAG fusion protein and the D53-GFP fusion protein in the leaves of *N. benthamiana*. (**b**) *In vivo* Co-IP analysis showed the interaction between SWR1-like-C-FLAG fusion proteins and D53-GFP fusion protein in the leaves of *N. benthamiana*. The non-interaction between SWR1-C-FLAG and D53-GFP was used as a negative control. *SWR1*-like genes used were derived from the following *Algae* species: *Chloropicon primus* (NCBI ID: UPR01476.1), *Chlorella sorokiniana* (NCBI ID: PRW61598.1), *Picochlorum sp*. BPE23 (NCBI ID: KAI8114589.1), *Chlorella ohadii*	(NCBI ID: KAI7845910.1), *Micromonas commoda* (NCBI ID: XP_002504538.1), *Micractinium conductrix* (NCBI ID: PSC74331.1), *Chlamydomonas eustigma* (NCBI ID: GAX77679.1), *Chlorella vulgaris* (NCBI ID: KAI3423408.1), and *Trebouxia sp*. A1-2 (NCBI ID: KAA6418788.1).

**Figure S23. Predicted three-dimensional structure and interaction interfaces of the TED1-D14-D53 complex by AlphaFold3.**

(**a**) Overall three-dimensional structure of the TED1-D14-D53 complex. TED1 is shown in dark blue, D14 in light blue, and D53 in magenta. ipTM = 0.28, pTM = 0.34. (**b**) Detailed interaction interface between TED1 and D14. Hydrogen bonds are formed between TED1^R1706^ and D14^S14^; between TED1^H1697^ and D14^S11^; between TED1^Y1709^ and D14^Q58^; and between TED1^R1708^ and both D14^N110^ and D14^D112^. Yellow dashed lines indicate hydrogen bond interactions. (**c**) Detailed interaction interface between TED1 and D53. Hydrogen bonds are formed between TED1^S1698^ and both D53^R113^ and D53^D153^; TED1^R1706^ also forms a hydrogen bond with D53^D153^. The hydrogen bond between TED1^R1706^ and D14^S14^ is also shown. Yellow dashed lines indicate hydrogen bond interactions.

**Table S1. The codon-optimized CDS sequence of *TED1***

ATGGCCAGCAAGGGCCCCAGATCCAAGCTGGACCATGAGACAAGAGCCAGACGGCAGAAGGCCCTGGAAGCTCCTAGAGGCGAGCCTCGGAGAACAAAGACCCACTGGGATCATCTGCTGGGCGAGATGGCCTGGCTGGCCAAAGAATTTGACGCCGAGCGGAAGTGGAAGCTGAGCATGGCCAAGAGAATCGCCCAGCGGGCTAACAAAGGCGTGGTGGATCAGGCCACCAAGGATGAGCGCAAGCAGAAAGAGGAAGAAGTCCGGCTGAGAAAGGTGGCCCTGAACATCTCCAAGGACGTGAAGAAGTTCTGGACCAAGATCGAGAAGCTGGTGTTCTACAAGAACCAGCTGGAACTGGAAGAACGGAAGAAGAAGGCTCTGGACAAGCAGCTGGACTTCCTGCTGGGACAGACCGAGCGGTACTCTACCATGCTGGCCGAGAACCTGGTGGATGTGCGGCTGCAGAATCAAGAGAACGACTCCCTGCAGACCAACCAGCGGTCCCAGCAAGAGCTGGCCCAAGAAAACATCAACGCCAGCTCTCCCACCGACGTGGACAACGTGGAAATCGACGACGACTACAACTCCAGCCTGGGCGAAGAACCCAAGGACGACGAGCACACCATCGACGAGGATGAGGCTCAGATCACCGAGGCCGAGAGAAACGAGGAACTGGCTGCTCTGCAGGCCGAGGCTGATCTGCCTCTGGACGATATCCTGAAGCTGTACACCAAGAACAAGGTGTCCAGAGAGTCCTCTCCTGACGGCCGGGACGTGTTCTCTGACTCCGACTCTAAGGACCTGATCAAGGACCCTCTGAACCAGGCCAACGGCTGCAACGACGAGTCTGACCACACCTCCTCTGACGAGGGCATCTCCTCTGAGGAAGCCGACGATTACCAGTCCTACTCCGAGTTCGTGAAGAAAAACACCGTGAAGTGCAACGGCAACATCTCCTCCGTGGACGCTAAGGACGATGAGGACTACGTGGCCAACGACGAAGGCAAGGATGACGAGGCTACCCTGTCCGAGGAAGAGGAACTCGCCAAGAAAGAAGATTGCGATCCCCTGGACGAAGTGAAGCTGCTGCAGAAAGAAAGCGAGATCCCCTTGGAGGAACTGCTGGCCAGATACCAGAAGGACGGCTACGCCGACGATGACACCACCGAGCTGGAAAACAGCCCTGCTCTGAGCGTGGAAGATGTGAACGCCAACATGCCCGTGGATGATGAGTCTGCCGATACCGTGGAAGTGAACCGGGATCTGTCTGCCGACACCATGAAGCTGACCAGAGATCAGTCCGCCGAGACAGTGAAAGTCAACAACGACCAGAGCGCCGAGATCGTGGAAGGCAACAATGACACCTTCGAGGACCACGAGTCCGCTGGAATGCTGGGTCCTGAACATGTGTCCGGCTCTGTGCTGCAGCTGGAAACCTCTGAGCCCATCGTGCAAGAGAACACCGCCAAAGAGGGCGACGTCACCGACACCAAGGCTATGGCCAATGGCGACAACTCCGACGTGATCGCCGATGCAGCTGCCGCTGCTAGATCTGCTCAGCCTACCGGCAACACCTTCTCCACCACCAACGTGCGGACCAAGTTTCCATTCCTGCTGAAGCACTCCCTGAGAGAGTACCAGCACATCGGCCTGGATTGGCTGGTGGCTATGTACGAGAAGCGGCTGAATGGCATCCTGGCCGATGAGATGGGCCTGGGCAAGACCATCATGACAATCTCCCTGCTGGCCCACCTGGCCTGCGAGAAAGGCATTTGGGGACCTCACCTGATCGTGGTGCCTACCTCCGTGATGCTGAACTGGGAGACAGAGTTCCTGAAGTGGTGCCCCGCCTTCAAGATCCTGACCTACTTCGGCTCTGCCAAAGAGAGAAAGCAGAAACGGCAAGGCTGGATGAAGCCCAACTACTTCCACGTGTGCATCACCACCTACCGGCTGGTCATCCAGGACTCCAAGGTGTTCAAGCGGAAGAAGTGGAAATACCTGATCCTGGACGAGGCTCACCTCATCAAGAATTGGAAGTCCCAGCGGTGGCAGACCCTGCTGAACTTCAACTCCAAGCGGAGAATCCTGCTGACCGGCACACCTCTGCAGAACGACCTGATGGAACTGTGGTCCCTGATGCACTTCCTGATGCCTCACGTGTTCCAGAGCCACCAAGAGTTCAAGGACTGGTTCTGCAACCCCATCTCCGGCATGGTGGAAGGCCAGGACAAAGTGAACAAAGAAGTGATCGACCGGCTGCACAACGTGCTGCGGCCTTTCATTCTGCGGCGGCTGAAGAGAGATGTGGAAAAACAGCTGCCCCAGAAACACGAGCACGTGATCTACTGCCGGCTGTCTAGAAGGCAGCGGAACCTGTACGAGGACTTTATCGCCTCCTCCGAGACACAGGCCACACTGGCCTCTGGCAACTACTTTGGCATGATCTCCATCATCATGCAGCTGAGGAAAGTGTGCAATCACCCCGACCTGTTCGAGGGCAGACCTATCATCTCCAGCTTCGACATGGCCGGGATCAATATGCAGATCTCCTCCTCCGTCGGAATGGTGCTGGACAAGGGCCCATTCTCTCAGGTGGACCTGTCCGACATGAACTTCGTGTTCACCCAGAACGAGTACAACATGACCAGCTGGGAAGAAGATGAGGTCGCCGCCATCTTCAGCCCCAGCATTACCCTGAGAGGCTCCGGCATCTCTCGGTCTACCAACGACGGCCAGAGATCCAACGGCACCAACATCTTTGAAGAGATCCACAACTCCCTGTGGGAAGAGAGAATCAAAGAGGCCAACGAGAGAGCCGCCTCTATCGCCTGGTGGAACAGAGTGCGGTGCCAGAAAAGACCCGTGTATGGCACCAATATCAGAGAGGTGCTGACCATCAAGCACCCCGTGTCTGACATCCTGGAAAAGAAGAAGAACCCTCTCTGCCACATGGAATTTTCCAGCAGCCTGGCCAACCTGGTGCTGTCCTCTGTGGACCGGTTCAAGACAATGCTGGATTTCATCGAGTCCTTCACCTTCGCTATCCCTGCCGCTAGAGCACCCGCTCCTCTGTTCTGGTGTAACAAGGGCAAGTCCCCTGTGCTGATCGAGCCTGCCTACCGCGAGAAGTGCATGAACGAGTTCAGCCCCGTGTTCTCCCCTATCCGGCCTGCTATTGTGCGGAGACAGGTGTACTTCCCCGACAGACGGCTGATCCAGTTCGACTGCGGAAAGCTGCAAGAACTGGCCATCCTGCTGCGGAGACTGAAGTCTGAGGGACACAGAGCCCTGATCTTTACCCAGATGACCAAGATGCTGGACATCCTCGAAGAGTTCATCAACCTGTATGGCTACACCTACCTGCGGCTGGACGGCTCTACACAGCCCGAAGAAAGACAGACACTGATGCAGCGGTTCAATACCAATCCTAAGTTCTTCCTGTTCATCCTGTCCACCAGATCCGGCGGAGTGGGCATCAATCTCGTGGGCGCTGATACCGTGATCTTCTACGACAGCGACTGGAACCCCGCTATGGATCAGCAGGCCCAGGACAGATGCCACAGAATCGGCCAGACACGCGAGGTGCACATCTACAGACTGATCAGCGAGTCCACCATCGAGGAAAACATCCTGAAGAAGGCCAACCAGAAGAGAGCACTGGACGACCTCGTGATCCAGCGGGGCTCTTACAACACCGAGTTCTTCAAGAAACTGGACCCTATGGAATTTTTCAGTGGCCACAGCTCCCTGCACGCCGAGAATCAGCAGAAGGACTGCTCTCTGAGCGCTGGCCCTAGCAACGGCACAAATCTGGCCCTGTCTAACGTGGACGTGGAAGCCGCTATCAGACAGGCTGAGGACGAGGCCGATTACATGGCTCTGAAGAGACTGGAACAAGAGGAAGCTGTGGACAATCAAGAGTTCTCCGAAGAGGCCGCTGGCCGGCTGGAAGAGGATGATCTGGTCAACGAGGACGATACCAAGCCTGACGAGCATACCAATGAGGAACACAAGTACCAGTGCTCCGATCTCGTGAAGGACAAGCACGTGGCACTGTCCATCAATCAGCTGGATGAGGAAAAGGCTATCACACTGGCTGGCGGAGATGGCGACATCGACATGCTGGCTGACGTGAAGCAGATGGCCGCTGCCGCAGCAGCCGCTGGACAGGCTTCTAGCTCTTTCGAAAACCAGCTGCGGCCCATCGACAGATACGCCATGCGGTTTCTTGAGCTGTGGGACCCCATCATCGACAAGGCCGCCATCAACTACCAAGTGAACGTGGAAGAGGAAGAGTGGGAACTCGAGCGGATTGAGAAACTGAAAGAGGATCTGGAAGCAGAGATCGATGAGGATCAAGAGCCCCTGTCCTACGAGTCCTGGGACGTTGACTTTGCCACCACCGCCTACAGACAGCATGTGGAAGCTCTGGCCCAGAAGCAGCTGTTCGAAGAACAAGAGCGGCAGGCCAGAGAGGCTGCTAAAGAGCTGGAAGAAAAGAACGACAACAGCACCCAGCGCAAGAAGTCCAAGAAGAACAAAAAGAAGGCCGCTAAGTTCAAGTCCCTCAAGAAGGGCAGACTGTCTAGCGAGAGCGAAGTGATGGTCGAAGAGACATCCGTGGACACCATGTCTATCGACGACAACGCTCCATCTCCTGAGCTGATGTCTGACGAGAGCGCCCACCACCACTCCAACAAGCACAAGCGGATCATGAGCACCAACGAGGAAGAAAACTCCAACTCTCGGTCCCTGAAAAAGCTGAAGAAAGCCCCTAAGAGCAGCTTCTCTAGCGAGGCTCTGTCCCCTAAGCACTTCCTCGAGGGAAAGCAGCTGAAGCTGAAGGACGAGCTGAACGACTCTGACCCCAAGTCCGGCGCCAGGATCAAGTCCGATGGCAGAATCTCCGTGCCTTGCATGCCTGTGAAGCGCGTGATGGTCATCAAGCCCGAGAGGCTGAAAAAGAAATCCCTGTGGTCCAGAGACTGCGCCTCCGATTCTTGGACCACTGAAGAGGACGCCGTGCTGTGTGCCACCGTGAATGAGTATGGACCCCTCTGGGAACTTGCCTCCGACAGCTTGCATTCTGTGCCTGGCGGCGCTTTCTACCGGGGCAGATATAGACACCCTGTGCACTGCTGCGAGAGATTCCGGGAACTCGTGTGCAAGCACATCCTGAGCGCCACCGACAACTCTAACTCCGAGAAGGTGCCATCCGGCACCGGCAAGGCCATTCTGAAGGTGTCCGAAGATCAGACCCAGATGCTGCTGAACGTGATCTCCGAGCTGCCCAACAACGAGCTGCTCCTGCAGAAACACTTCATGGCCGTGCTGAGTTCCGTGTGGCGGTCTAAGTCTGCCTGCGAGTCTCACTGCGTGATGTCCTACAGCAATACCCTGCAGAAGCCTGGCAGGCTGAGCGAGAACTGGTCCATGACCAACTTCCGGCCTAACTTCAACCTCGTGCGCACCGCTTTGGCTGATGCCCAGGTTCAGTGCCCTAGAATGGTGGTGCCAACCAGCAACCACGAGAGCAGACGGAACTTCCTGGAACTTGAGCTGGATTTTCTGACCGACCGGGACGACTACGAGGCTGACTTTCCTTCCGTGGTCAACGTGTCCATCCTCGAGCCTGAGCCTCTGAAGCACGCTGTGGAACCCGTGGAACACAGCCTGCTGAGCACCCTGTCTTACCGGCACGCTGAGAACAGATTCAGGATGGTGTCCGAGACATGCTTCGAAGGCGAGGGCTCTCACTGGGCCTCTAGCGCTTTTCACACCTACGATGCCGGCAGACATAAGAGCGGCCCTAAGTCCATCGGAAAGCACAAGGCCTCCAGCGAGTCTGGCAGACCTCCTAAGTCTAAGATCCAGAGAACAACCGAGCCTCAAGAGGTGCCCGTGACCAACAACTTTCACAGAATCCCCGGCCAGCTCCTGCACAACTCTGCCGAGTTTCACATCACCCAGTCTCTGTCCGACCTGGGAATCTCTGATTCCGAGTTCACCTACTTTGACAACCTGCCACAAGAGGCCGAAACCGAGTTTGTGCCCTACCAGTACGATTCCGACGTGCTGAGCGGCATCGAAGAACTTGACCCACTGACCGACTTTACCGACATCGGATGA

**Table S2. Target sites used for CRISPR/Cas9 knockout**

| Primer name | Primer sequence (5' to 3') |
| --- | --- |
| *TED1*-1 | TAAAGCTGCTATAAACTATCAGG |
| *TED1*-2 | CCGTGAAGCAGCAAAAGAGTTGG |
| *D27* | GAATGCAGCAAAGTACTCCCTGG |
| *D17* | TCCCCGGAGACCGAGGCGGGCGG |
| *D10* | GAAGTGGAACGTCACGAACGG |
| *D3* | AGATCTCAATTAAGGTGGAGG |
| *D14* | GGACGACGACGCAGGGGGCGCGG |
| *D53* | AGCAGGGCCAGCGGTGGCGGCGG |
| *H2AV1* | GCGGGAGGAGCGGGAGACGGG |
| *H2AV2* | GCGGGAGGAGCGGGAGACGGG |
| *H2AV3* | CTTGCTCGCCGCGAAGACGACGG |

Note: PAM, protospacer adjacent motif, is marked in red letters.

**Table S3. Primers used for construction of vectors**

| Primer name | Primer sequence (5' to 3') |
| --- | --- |
| pCAMBIA2300-TED1-F | GGATCCTCTAGAGTCGACATGGCATCAAAAGGTCCTCG |
| pCAMBIA2300-TED1-R | GCATGCCTGCAGGTCGACTCCGATGTCTGTGAAATC |
| pCAMBIA2300-3×Flag-D14-F | GGATCCTCTAGAGTCGACATGCTGCGATCGACGCAT |
| pCAMBIA2300-3×Flag-D14-R | GCATGCCTGCAGGTCGACTTATTTCGAACCCGGGGTACC |
| pCAMBIA2300-PIE1-F | GGATCCTCTAGAGTCGACATGGCGTCTAAAGGTGGTAA |
| pCAMBIA2300-PIE1-R | GCATGCCTGCAGGTCGACCTACTCTATTTCTGAGATAT |
| pCAMBIA2300-SWR1-F | GGATCCTCTAGAGTCGACATGACCACATCTCGTAAATCG |
| pCAMBIA2300-SWR1-R | GCATGCCTGCAGGTCGACTCAATAATAATAACCGTTGGCA |
| pCAMBIA1300-3×Flag-TED1-C-F | AACACGGGGGACTCTAGAATGGAATTTTCATCAAGCCT |
| pCAMBIA1300-3×Flag-TED1-C-R | CTTGTAGTCCATGTCGACTCCGATGTCTGTGAAAT |
| pCAMBIA1300-3×Flag-TED1-NM-F | AACACGGGGGACTCTAGAATGGCATCAAAAGGTCCTCGA |
| pCAMBIA1300-3×Flag-TED1-NM-R | CTTGTAGTCCATGTCGACAATAAAATCAAGCATTGT |
| pCAMBIA1305GFP-TED1^CO^-F | GCCCAGATCAACTAGTATGGCCAGCAAGGGCCCCAGAT |
| pCAMBIA1305GFP-TED1^CO^-R | TGCTCACCATGGATCCTCCGATGTCGGTAAAGTCGGTC |
| pCAMBIA1305GFP-D14-F | TGGACGAGCTGTACAGATCTATGCTGCGATCGACGCAT |
| pCAMBIA1305GFP-D14-R | GGGCGGCCGCTTTAAGATCTTTAGTACCGGGCGAGAGC |
| pCAMBIA1305GFP-D53-F | GCCCAGATCAACTAGTATGCCCACTCCGGTGGCC |
| pCAMBIA1305GFP-D53-R | TGCTCACCATGGATCCACAATCTAGAATTAT |
| pCAMBIA1305GFP-D3-F | GCCCAGATCAACTAGTATGGCGGAAGAGGAGGAG |
| pCAMBIA1305GFP-D3-R | TGCTCACCATGGATCCATCATCAATTTGCCGGCT |
| pAN580-TED1^CO^-F | CGGAGCTAGCTCTAGAATGGCCAGCAAGGGCCCCAGAT |
| pAN580-TED1^CO^-R | TGCTCACCATGGATCCTCCGATGTCGGTAAAGTCGGTC |
| P2YC/N-D14-F | CATTTACGAACGATAGTTAATTAAATGCTGCGATCGACGCAT |
| P2YC/N-D14-R | CACTGCCACCTCCTCCACTAGTGTACCGGGCGAGAGC |
| P2YC/N-D53-F | CATTTACGAACGATAGTTAATTAAATGCCCACTCCGGTGGCC |
| P2YC/N-D53-R | CACTGCCACCTCCTCCACTAGTACAATCTAGAATTAT |
| P2YC/N-TED1^CO^-F | CATTTACGAACGATAGTTAATTAAATGGAATTTTCATCAAGCCT |
| P2YC/NTED1^CO^-R | CACTGCCACCTCCTCCACTAGTTCCGATGTCTGTGA |
| pGEX4T-1-D53-F | TGGATCCCCGGAATTCATGCCCACTCCGGTGGCC |
| pGEX4T-1-D53-R | GTCGACCCGGGAATTCTCAACAATCTAGAATTAT |
| pET-28a-D14-F | AATGGGTCGCGGATCCATGCTGCGATCGACGCAT |
| pET-28a-D14-R | GACGGAGCTCGAATTCTTAGTACCGGGCGAGAGC |
| pET-28a-D3-F | AATGGGTCGCGGATCCATGGCGGAAGAGGAGGAG |
| pET-28a-D3-R | GACGGAGCTCGAATTCCTAATCATCAATTTGCCGGCT |
| pMal-c2X-TED1-C-F | TTCAGAATTCGGATCCATGGAATTTTCATCAAGCCT |
| pMal-c2X-TED1-C-R | CGACTCTAGAGGATCCTCATCCGATGTCTGTGAAATC |
| pET-28a-TED1^CO^-F | AATGGGTCGCGGATCCATGGCCAGCAAGGGCCCCAGAT |
| pET-28a-TED1^CO^-R | GACGGAGCTCGAATTCCTATCCGATGTCGGTAAAGTCGGTC |
| pGEM-T Easy-TED1-F | ATCGAAGTCTGCCTGTGAGT |
| pGEM-T Easy-TED1-R | AAAAGTTACGGCGGGACTCA |

Note: The adapter sequences in the primers are labeled in red.

**Table S4. Primers used in Real-time Quantitative PCR**

| Primer name | Primer sequence (5' to 3') |
| --- | --- |
| qRT-TED1-F | AAGAATGGGAACTTGAACGC |
| qRT-TED1-R | ACATGTTGCCGATATGCTGT |
| qRT-D27-F | TCTGGGCTAAAGAATGAAAAGGA |
| qRT-D27-R | AGAGCTTGGGTCACAATCTCG |
| qRT-D17-F | CGATCCCACTTCACTTTCTACGAG |
| qRT-D17-R | GATGTAGTGGGTGTCGGTGAAG |
| qRT-D10-F | GCAAGACGTTCGAGGAGGT |
| qRT-D10-R | TTACTTGCTGTTCCTTTTCCTGG |
| qRT-D3-F | GCTTTTCATCCATGGCACCA |
| qRT-D3-R | GATCATTCTCTGGGGCTGGA |
| qRT-D14-F | CGCCTTCGTCGGCCACTC |
| qRT-D14-R | TCGAACCCGCCGTGGTAGTC |
| qRT-D53-F | GAGGAGGATAGGAAACCTG |
| qRT-D53-R | TTTCACTGCTGGTACTGTGTC |
| qRT-FC1-F | ATTCGCGGCACTACTGCTGATG |
| qRT-FC1-R | TTAGTGCCGTACCGTTCGTTCG |
| qRT-PIE1-F | CACTCCTAAATGTTGCAGCTGA |
| qRT-PIE1-R | TGGAGAGCGGATTCTAACAACT |
| qRT-SWR1-F | ACGAAAGCTTGCACGAATACAT |
| qRT-SWR1-R | TTGTCATACTTCAATGCGCCAT |
| qRT-Ubiquitin-F | ACCCTGGCTGACTACAACATC |
| qRT-Ubiquitin-R | AGTTGACAGCCCTAGGGTG |

**Table S5. Primers used for the yeast two-hybrid assay**

| Primer name | Primer sequence (5' to 3') |
| --- | --- |
| AD-TED1-N-F | GGAGGCCAGTGAATTCATGGCATCAAAAGGTCCTCGA |
| AD-TED1-N-R | TCATCTGCAGCTCGAGTTACGCTTTAGTATCAGTTAC |
| AD-TED1-M-F | GGAGGCCAGTGAATTCATTCTCAAGTTGTACACTAA |
| AD-TED1-M-R | TCATCTGCAGCTCGAGTTAAATAAAATCAAGCATTGT |
| AD-TED1-C-F | GGAGGCCAGTGAATTCATGGAATTTTCATCAAGCCT |
| AD-TED1-C-R | TCATCTGCAGCTCGAGTCATCCGATGTCTGTGA |
| BD-D14-F | CATGGAGGCCGAATTCATGCTGCGATCGACGCAT |
| BD-D14-R | TAGTTATGCGGCCGCTGCAGTTAGTACCGGGCGAGAGCG |
| AD-TED1-C1-F | GGAGGCCAGTGAATTCGAATTTTCATCAAGCCT |
| AD-TED1-C1-R | TCATCTGCAGCTCGAGTCAAAGCATCTTAGTCATCTG |
| AD-TED1-C2-F | GGAGGCCAGTGAATTCGATATACTGGAGGAGTTC |
| AD-TED1-C2-R | TCATCTGCAGCTCGAGTCAACCTATCCTGTGGCATCT |
| AD-TED1-C3-F | GGAGGCCAGTGAATTCCAAACTCGTGAAGTTCAC |
| AD-TED1-C3-R | TCATCTGCAGCTCGAGTCAATCAATTATTGGATCCCA |
| AD-TED1-C4-F | GGAGGCCAGTGAATTCAAAGCTGCTATAAACTAT |
| AD-TED1-C4-R | TCATCTGCAGCTCGAGTCACTTCGCTGCCTTCTTCTT |
| AD-TED1-C5-F | GGAGGCCAGTGAATTCTTCAAATCTTTGAAGAAA |
| AD-TED1-C5-R | TCATCTGCAGCTCGAGTCATCTGCCAGGTTTCTGGAG |
| AD-TED1-C6-F | GGAGGCCAGTGAATTCCTCAGCGAGAACTGGTCC |
| AD-TED1-C6-R | TCATCTGCAGCTCGAGTCATCATCCGATGTCTGTGAA |
| BD-AtD14-F | CATGGAGGCCGAATTCATGAGTCAACACAACATCTT |
| BD-AtD14-R | TAGTTATGCGGCCGCTGCAGTCACCGAGGAAGAGCTCGCC |
| AD-PIE1-C1-F | GGAGGCCAGTGAATTCTACATGTATTCATCAATCCT |
| AD-PIE1-C1-R | TCATCTGCAGCTCGAGCTAAAGCATCTTGGTCATTTGCG |
| AD-PIE1-C2-F | GGAGGCCAGTGAATTCGACGTCTTGGAGGCTTTCAT |
| AD-PIE1-C2-R | TCATCTGCAGCTCGAGCTAACCTATCCTGTGGCAGCGAT |
| AD-PIE1-C34-F | GGAGGCCAGTGAATTCCAGACACGTGAAGTGCATAT |
| AD-PIE1-C34-R | TCATCTGCAGCTCGAGCTAAGCTTCTTCTTGTTCTACTC |
| AD-PIE1-C5-F | GGAGGCCAGTGAATTCGTGGACAATCAAGAGTTCAC |
| AD-PIE1-C5-R | TCATCTGCAGCTCGAGCTAGGGAACACAATCTCTAGACC |
| AD-PIE1-C6-F | GGAGGCCAGTGAATTCTCGCCTGATTCTTGGTTGCC |
| AD-PIE1-C6-R | TCATCTGCAGCTCGAGCTACTACTCTATTTCTGAGATAT |
| AD-SWR1-C1-F | GGAGGCCAGTGAATTCGATAAATCAAGCATTATTGA |
| AD-SWR1-C1-R | TCATCTGCAGCTCGAGTCACAAAACTTTTGTCATTTGTG |
| AD-SWR1-C2-F | GGAGGCCAGTGAATTCGACGTTCTGGAACAGTTTTT |
| AD-SWR1-C2-R | TCATCTGCAGCTCGAGTCATCCAATTCTGTGACATCTAT |
| AD-SWR1-C3-F | GGAGGCCAGTGAATTCCAGACTCGAGATGTGCATAT |
| AD-SWR1-C3-R | TCATCTGCAGCTCGAGTCAATAATAATAACCGTTGG |

Note: The adapter sequences on the primers are labeled in red.
